# Relationship between lead exposure and different types of hypertension: systematic review and dose–response meta-analysis

**DOI:** 10.3389/fpubh.2025.1706805

**Published:** 2025-12-10

**Authors:** Peilin Yang, Tianyi Ji, Zhendong Yang, Jiayuan Song, Hongwei Liu, Haixia Fan

**Affiliations:** 1 Heilongjiang University of Chinese Medicine, Haerbin, China; 2 College of Integrative Medicine, Changchun University of Chinese Medicine, Changchun, China; 3 Department of Neurology, Taiyuan City Central Hospital, The Ninth Clinical Medical College of Shanxi Medical University, Taiyuan, Shanxi Province, China; 4 Department of Sleep Center, First Hospital of Shanxi Medical University, Taiyuan, Shanxi Province, China

**Keywords:** lead exposure, hypertension, meta-analysis, systematic review, dose–response relationship, risk factor

## Abstract

**Background:**

This systematic review and meta-analysis aimed to quantify the association between environmental and occupational lead exposure and the risk of various hypertension subtypes. We further evaluated this relationship through a dose–response analysis to provide a scientific basis for targeted public health interventions.

**Methods:**

Observational studies on lead exposure and hypertension were searched from Chinese/English databases (inception to June 9, 2025). Two researchers independently screened studies, extracted data, and assessed bias risk. Random-effects meta-analysis calculated pooled ORs (95% CIs); meta-regression/subgroup analyses explored heterogeneity. Egger’s test evaluated publication bias, sensitivity analyses verified result robustness, and dose–response analysis was applied to multi-level exposure data.

**Result:**

24 studies (181,500 participants) were included. Lead exposure correlated significantly with hypertension (pooled OR = 1.27, 95% CI: 1.20–1.34) with high heterogeneity (I^2^ = 91.4%, *p* < 0.001). Stronger associations were found for blood lead (OR = 1.32), essential hypertension (OR = 1.31), and resistant hypertension (OR = 1.36); no significant associations for bone lead or gestational hypertension. Hypertension risk rose sharply at blood lead ≥ 0.107 μg/dl, reaching OR = 4.85 at 8.435 μg/dl. Potential publication bias existed, but sensitivity analyses confirmed robustness.

**Conclusion:**

Lead exposure is a hypertension risk factor with a clear dose–response relationship. It is recommended to include blood lead in hypertension risk assessment and set a blood lead action level of < 0.107 μg/dl for high-risk groups to reduce hypertension burden from lead pollution.

**Systematic review registration:**

https://inplasy.com/inplasy-2025-9-0008/.

## 1 Introduction

As one of the most prevalent chronic non-communicable diseases globally, hypertension is a major risk factor for cardiovascular disease, stroke, and kidney disease, imposing a substantial burden on public health systems (1). According to the World Health Organization, the global prevalence of hypertension is rising, with an observable trend towards a younger age of onset. Its pathogenesis is complex, involving intricate interactions among genetic, environmental, and lifestyle factors (2). Consequently, the association between environmental pollutant exposure and hypertension has garnered increasing research attention. Among these pollutants, lead, a ubiquitous environmental toxicant, is of particular scientific interest due to its potential adverse effects on the cardiovascular system.

Lead is a heavy metal with established neurotoxic and hematotoxic properties that enters the human body through pathways such as air, water, food, and soil. Its chronic accumulation can result in damage to multiple organ systems (3, 4). While previous epidemiological studies suggest an association between lead exposure and an increased risk of hypertension, the strength of this link remains controversial (5, 6). Some studies have reported significant positive correlations between biomarkers of lead exposure—such as blood lead and bone lead—and hypertension (7, 8), while others have failed to observe a clear relationship (9). These discrepancies may stem from variations in baseline population characteristics (e.g., geographic region, age, genetic background), exposure assessment methods (e.g., blood lead vs. bone lead), study design (e.g., cross-sectional vs. cohort studies), and the extent of confounding control. Furthermore, it remains unclear whether a dose–response relationship exists between lead exposure and hypertension, and whether this relationship varies across hypertension subtypes—such as essential hypertension, resistant hypertension, and hypertension during pregnancy—has not yet been conclusively established.

Systematic reviews and meta-analyses are essential methodologies for synthesizing existing research evidence. By quantitatively combining results from multiple independent studies, they enhance statistical power, mitigate random errors inherent in individual studies, and facilitate the exploration of heterogeneity sources, thus providing more robust evidence for scientific inquiries (10). Although several meta-analyses have previously investigated the association between lead exposure and hypertension, most have not thoroughly examined variations across key subgroups (e.g., geographic region, study design, hypertension subtype). Moreover, a systematic assessment of the dose–response relationship remains limited, and the quantitative association between varying levels of lead exposure and hypertension risk has not been sufficiently elucidated (6, 11).

Based on the aforementioned research background, this study aims to comprehensively evaluate the association between lead exposure and different types of hypertension through systematic literature search and inclusion, using systematic review and dose–response meta-analysis methods. It further seeks to explore subgroup differences across various populations, study designs, and exposure characteristics. Additionally, by conducting dose–response analysis, the study intends to clarify the quantitative relationship between lead exposure levels and the risk of hypertension onset. The findings will provide scientific evidence for elucidating the role of lead exposure in the pathogenesis of hypertension and for developing targeted public health intervention strategies.

## 2 Materials and methods

### 2.1 Protocol and registration

To ensure methodological transparency and rigor, this meta-analysis was conducted and reported in strict accordance with the Preferred Reporting Items for Systematic Reviews and Meta-Analyses (PRISMA) 2020 statement (12, 13). These guidelines provide a standardized framework for conducting and reporting systematic reviews and meta-analyses. Additionally, the study protocol has been registered with the International Platform of Registered Systematic Review and Meta-analysis (registration number: INPLASY202590008. Specify the inclusion and exclusion criteria for the review and how studies were grouped for the syntheses), further enhancing transparency and minimizing the risk of bias.

### 2.2 Literature search strategy

We conducted a comprehensive search across four major databases—PubMed, Web of Science, Embase, EBSCO and Scopus—to identify all relevant studies on the relationship between lead exposure and hypertension. The search period spanned from the inception of each database to 9 June 2025. The terms combined controlled vocabulary (MeSH/Emtree) with free-text keywords covering “Lead,” “Lead Poisoning,” “Blood Lead,” “Bone Lead,” “Urinary Lead,” “Hypertension,” “Blood Pressure,” “Essential Hypertension,” “Resistant Hypertension,” “Gestational Hypertension,” and “Postpartum Hypertension.” These queries were further restricted to observational study designs (cross-sectional, cohort, case–control) and required the presence of multivariable-adjusted effect estimates (OR, RR, or HR with 95% CI). Detailed search strategies for each database are provided in Supplementary Tables S1–S5.

### 2.3 Inclusion criteria

To examine the association between lead exposure and hypertension, we included only observational studies that enrolled adults (≥18 years) and provided a clear diagnosis of hypertension—whether essential, resistant, gestational, menopausal, or uncontrolled—based on established clinical criteria. Studies were eligible regardless of geographic origin or design (cross-sectional, cohort, or case–control), provided that they reported multivariable-adjusted effect estimates (odds ratio, relative risk, hazard ratio) with corresponding 95% confidence intervals and explicitly quantified lead exposure using blood, bone, or urine biomarkers.

### 2.4 Exclusion criteria

The following studies were excluded for analysis: studies that did not involve patients with hypertension diagnosis; studies whose results do not meet the inclusion criteria; reviews, case studies, survey analysis, meeting summaries and unrelated literature; repetitive publications.

### 2.5 Literature screening and data extraction

Following predefined eligibility criteria, two reviewers (Yang and Ji) independently screened the titles, abstracts, and full texts of articles retrieved. Disagreements were resolved by re-examining the original articles and reaching consensus through discussion. The following data were extracted from each included study and compiled into Table 1: author names, publication year, country, study design (cross-sectional, cohort, or case–control), type of lead exposure (blood, bone, or urine lead), hypertension subtype (essential, resistant, gestational, menopausal, or uncontrolled), sample size, age, sex, effect measure (odds ratio), 95% confidence interval (lower–upper CI), grouping basis, and covariate adjustments (alcohol, smoking, physical activity, BMI, etc.).

**Table 1:** Basic characteristics of included studies and summary of exposure information.

Study	Model correction adjustment	Country	Research design	Age	Population	Types of lead	Types of hypertension	Grouping basis	Quality score
W.L.A.M. de Kort (1987) (53)	/	Netherlands	cross-sectional study	Exposure group: 42.1 ± 1.2; control group: 38.2 ± 1.1	105	Blood lead	Hypertension		9
Michael Rabinowttz (1987) (54)	/	The United States	cross-sectional study	28 ± 5	3,210 (females)	Blood lead	Hypertension		5
Howard Hu (1996) (55)	Gender, age, BMI, family history of hypertension, years of smoking, ethanol intake, dietary sodium and dietary calcium intake.	The United States	case–control study	66.6 ± 7.2	590	Tibia bone lead level	Hypertension		9
Stephen J. Rothenberg (2002) (56)	Data were adjusted for postpartum hypertension, education level, immigration status, current smoking status, number of births, age, and BMI.	The United States	cohort study	15–44	667	blood lead	Hypertension In Pregnancy	Third trimester	7
								Postpartum	
						Tibia lead		Third trimester	
								Postpartum	
						Calcaneus lead		Third trimester	
								Postpartum	
David Martin (2006) (57)	Age, gender, BMI, sodium intake, potassium intake, homocysteine level, time of day, and test technician, socioeconomic status, race/ethnicity.	The United States	cross-sectional study	50–70	964	Blood lead	Hypertension		8
						Tibia lead			
Iman Al-Saleh (2006) (58)	/	Saudi arabia	case–control study	45–93	185	Blood lead	Hypertension		7
Chadi Yazbeck (2009) (59)	Maternal age, blood levels of cadmium, manganese, and selenium, hematocrit, parity, BMI, gestational diabetes, education level, socioeconomic status, place of residence, and smoking during pregnancy.	France	cohort study	18–45	971 (females)	Blood lead	Pregnancy-Induced Hypertension		8
Byung-Kook Lee (2016) (60)	Factors such as gender, age, area of residence, level of education, smoking and drinking status, BMI, physical activity, serum creatinine and hemoglobin	South korea	cross-sectional study	≥19	11,979	Blood lead	Hypertension	Female	8
							Hypertension	Male	
Angela Gambelunghe (2016) (61)	Sex, age, smoking, drinking, waist circumference and education level	Sweden	cohort study	46–67	Baseline: 4,452 Follow-up: 2,904	Blood lead	Hypertension		8
Alexander R. (2018) (62)	BMI, age, smoking, annual income, education level, racial factors, family history of hypertension.	The United States	cohort study	48–93	475	Tibia models	Resistant Hypertension	Tibia models	7
						Patella models		Patella models	
						Blood models		Blood models	
Wen-Yi Yang (2018) (63)	Age, BMI, covariate heart rate, hip-to-waist ratio, smoking, total cholesterol to high-density lipoprotein cholesterol ratio, g-glutamyl transferase, and estimated glomerular filtration rate.	The United States	cross-sectional study	28.6 (average age)	236 (males)	Blood lead	Hypertension	Office hypertension	7
								24-h hypertension	
								Awake hypertension	
								Asleep hypertension	
								White coat effect	
Tiange Liu (2019) (64)	Delivery age, race, education level, parity, pre-pregnancy BMI, smoking status during pregnancy	The United States	cohort study	28 (average age)	1,274	Blood lead	preeclampsia Hypertension		8
Min Gi Kim (2019) (65)	Total cholesterol and BMI adjusted for age in 2000 (all continuous variables).	South korea	cohort study	31.1 ± 8.5	7,341	Blood lead	Hypertension		5
Katherine M. Johnson (2020) (66)	Maternal age and parity	The United States	cross-sectional study	34 (median age)	98	Bone lead	Hypertensive disorders of pregnancy		7
Hui Miao (2020) (67)	Age, sex, race, proportion of household income to poverty, level of education, smoking status, serum cotinine, alcohol intake, BMI, menopausal status	The United States	cross-sectional study	≥20	30,762 (15,679 males; 15,083 females)	Blood lead	Uncontrolled Hypertension	Male	8
							Uncontrolled Hypertension	Women	
Su Zhen Wu (2021) (68)	Age of delivery, pre-pregnancy BMI, parity, mode of conception and level of education	China	cohort study	29.04 ± 4.25	2,174 (females)	Blood lead	preeclampsia Hypertension	Severe Preeclampsia	8
								Mild Preeclampsia	
Hai Duc Nguyen (2021) (69)	Age, hypertension treatment, log2 sodium intake, dyslipidemia, area of residence, smoking status, certified smokers, drinking status, level of education, occupation, physical activity, and family history of cardiovascular disease, diabetes or hyperlipidemia.	South korea	cross-sectional study	≥18	7,226 (females)	Blood models	hypertension during menopause	Premenopausal women	8
								Postmenopausal women	
Ziyao Huang (2022) (70)	Adjusted for age, sex, race, education level, household income to poverty ratio, BMI, alcohol use and smoking behavior	The United States	cross-sectional study	20–85	32,289	Blood lead	Hypertension		7
Jeoung A. Kwon (2023) (71)	Age, sex, education, monthly household income, employment status, smoking status, alcohol consumption, metal content of natural logarithm conversion, BMI, prevalence of diabetes and survey year	South korea	cross-sectional study	≥19	8,510 (4,259 males and 4,251 females)	Blood lead	Hypertension	Inactive	8
								Moderate active	
								Most active	
Songfeng Zhao (2023) (72)	According to age, gender, race, education level, PIR, BMI, serum cotinine, alcohol intake, all other metals.	The United States	cross-sectional study	≥20	5,092	urine lead	Hypertension		8
Yuqing Huang (2023) (73)	Age, gender, education level, ethnic background, poverty to income ratio, marital status, smoking and drinking habits, BMI, glomerular filtration rate, diabetes, stroke and cardiovascular disease and other factors.	The United States	cross-sectional study	≥18	20,073	Blood lead	Hypertension		7
Hao Chen (2023) (74)	Age, race, education level, marital status, annual income, BMI, smoking, drinking, chronic kidney disease, glomerular filtration rate, diabetes mellitus	The United States	cross-sectional study	≥18	38,281	Blood lead	Resistant Hypertension	Male	7
								Female	
								≤60 years	
								>60 years	
								Kidneydysfunction	
								Nokidneydysfunction	
							Hypertension	Male	
								Female	
								≤60 years	
								>60 years	
								Kidneydysfunction	
								Nokidneydysfunction	
Arturo Corbaton’Anchuelo (2024) (75)	Age, gender, BMI, diabetes and creatinine clearance rate.	Spain	cross-sectional study	21–80	73	Blood lead	Resistant Hypertensive		6
Cuixiao Wang (2024) (76)	Confounding factors such as age, sex, race, BMI, PIR, education level, smoking status, drinking status, marital status, diabetes, coronary heart disease, stroke, and blood metal levels.	The United States	cross-sectional study	≥18	4,473	Blood lead	Hypertension		7

### 2.6 Quality assessment

In this study, three quality evaluation tools were used according to the type of study design: 1 Case–control study using the Newcastle-Ottawa Scale (NOS) to assess whether the case definition is clear, case representation, control selection and definition, exposure confirmation, comparability and result evaluation. NOS was also used in the cohort study, focusing on the representativeness of the exposed cohort, non-exposed cohort selection, exposure confirmation, baseline no outcome, comparability, outcome evaluation, follow-up time adequacy and cohort follow-up integrity. The cross-sectional study used the JBI (Joanna Briggs Institute) 9-item list to conduct a comprehensive score from the perspectives of sample framework, sampling method, sample size, research object and scene description, data coverage, disease identification method, measurement standard consistency, statistical method and response rate. Each item takes ‘1 ‘as the full score, and the higher the total score, the better the research quality.

### 2.7 Statistical analysis

We conducted a meta-analysis using STATA 18.0 software, following these steps: First, we selected the odds ratio (OR) as the effect-size measure and calculated its 95% confidence interval (CI). Heterogeneity was then examined with *p* values and I^2^ statistics; when *p* > 0.1 and I^2^ ≤ 50% we applied a fixed-effect model (FE), whereas *p* ≤ 0.1 and I^2^ > 50% prompted a random-effects model (RE) (14). Publication bias was evaluated with funnel plots and Egger’s test (15), and sensitivity was explored by sequentially excluding individual studies (16). In addition, we carried out meta-subgroup analyses to identify sources of heterogeneity. Finally, We conducted subgroup analyses and meta-regression to investigate potential sources of heterogeneity in the pooled effect estimates (17).

## 3 Result

### 3.1 Overall results

**Figure 1:**
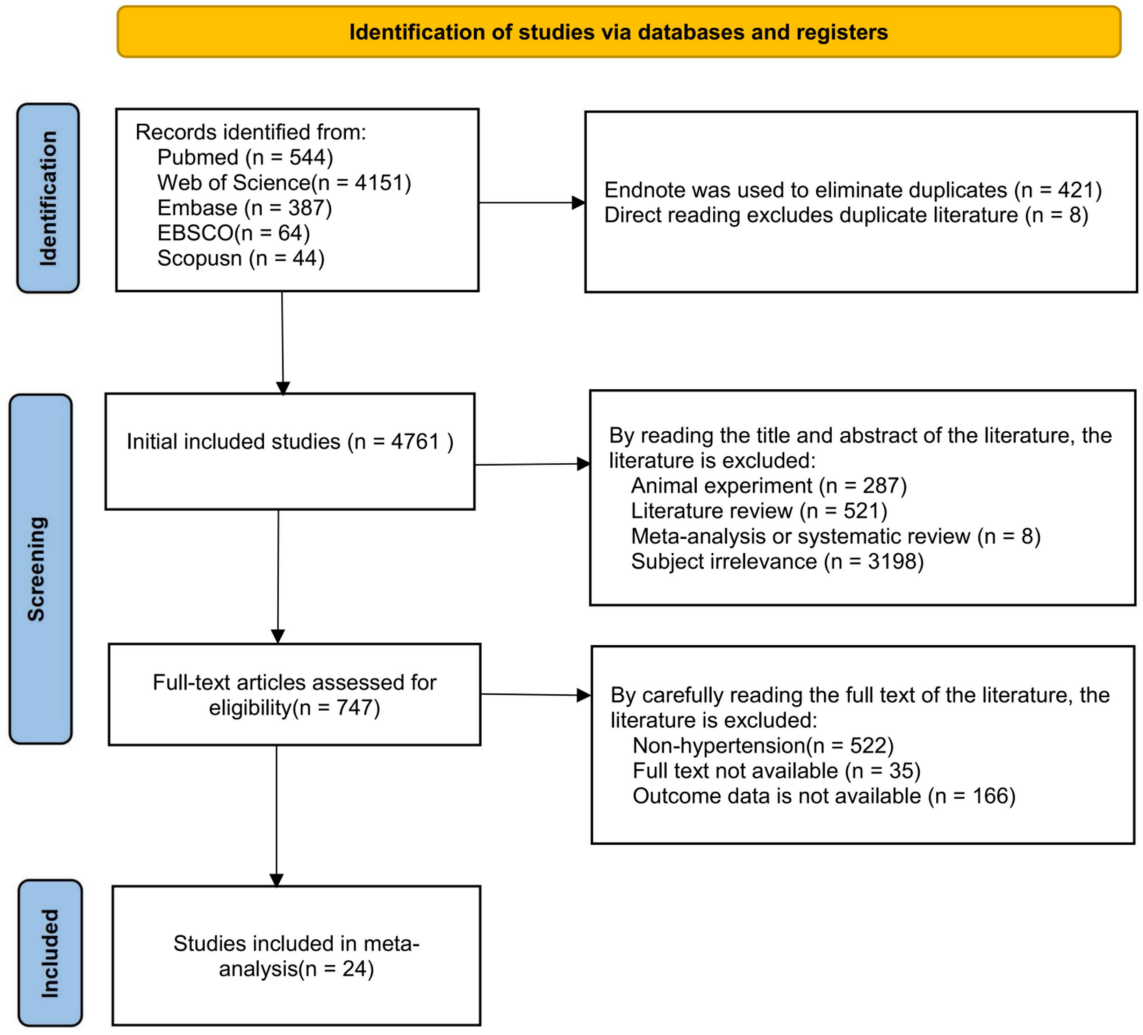
Study selection flow chart.

**Figure 2:**
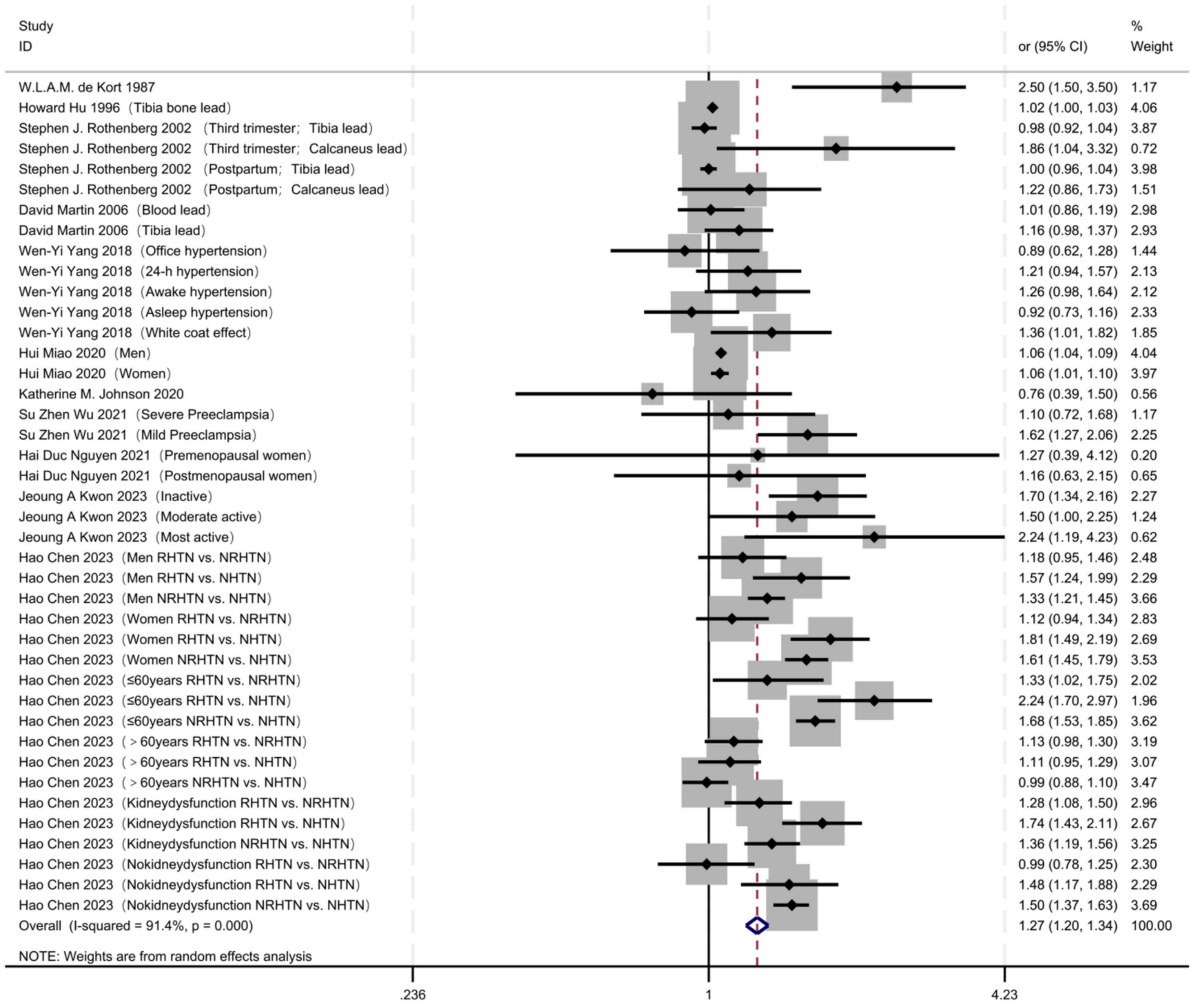
Total analysis: forest plot of lead exposure and risk of hypertension.

A total of 5,190 articles were retrieved, and 24 articles were finally included. The retrieval process (Figure 1). A total of 24 studies were included (Table 1), comprising 181,500 individuals; among them, 11 studies reported the OR for the association between lead exposure and hypertension risk (Table 2). Seventeen studies reported the OR for hypertension risk when lead exposure was in the highest quantile (see Supplementary Table). Five studies provided dose–response data relating blood lead levels to hypertension risk (i.e., changes in hypertension incidence with increasing blood lead exposure) (Table 3). We pooled the 11 studies reporting ORs for the association between lead exposure and hypertension; the overall analysis revealed a significant association, with hypertension risk increasing markedly as lead exposure rose (OR = 1.27, 95% CI: 1.20–1.34, I^2^ = 91.4%, *p* < 0.001) (Figure 2).

**Table 2:** Baseline table of correlation between lead exposure level and hypertension.

Study	OR	LCI	UCI	Subgroup (country)	Subgroup (research design)	Subgroup (types of lead)	Subgroup (types of hypertension)	Model adjustment type			
								drinking alcohol	smoking	physical activity	BMI
W.L.A.M. de Kort (1987)	2.5	1.5	3.5	Netherlands	cross-sectional study	Blood lead	Hypertension	/	/	/	/
Howard Hu (1996) (Tibia bone lead)	1.019	1.004	1.035	The United States	case control study	Bone lead	Hypertension	YES	NO	NO	YES
Stephen J. Rothenberg (2002) (Third trimester; Tibia lead)	0.98	0.92	1.04	The United States	cohort study	Bone lead	Hypertension In Pregnancy	NO	YES	NO	yes
Stephen J. Rothenberg (2002) (Third trimester; Calcaneus lead)	1.86	1.04	3.32	The United States	cohort study	Bone lead	Hypertension In Pregnancy	NO	YES	NO	yes
Stephen J. Rothenberg (2002) (Postpartum; Tibia lead)	1	0.96	1.04	The United States	cohort study	Bone lead	Hypertension In Pregnancy	NO	YES	NO	yes
Stephen J. Rothenberg (2002) (Postpartum; Calcaneus lead)	1.22	0.86	1.73	The United States	cohort study	Bone lead	Hypertension In Pregnancy	NO	YES	NO	yes
David Martin (2006) (Blood lead)	1.01	0.86	1.19	The United States	cross-sectional study	Blood lead	Hypertension	NO	NO	NO	Yes
David Martin (2006) (Tibia lead)	1.16	0.98	1.37	The United States	cross-sectional study	Bone lead	Hypertension	NO	NO	NO	Yes
Wen-Yi Yang (2018) (Office hypertension)	0.89	0.62	1.28	The United States	cross-sectional study	Blood lead	Hypertension	NO	YES	NO	YES
Wen-Yi Yang (2018) (24-h hypertension)	1.21	0.94	1.57	The United States	cross-sectional study	Blood lead	Hypertension	NO	YES	NO	YES
Wen-Yi Yang (2018) (Awake hypertension)	1.26	0.98	1.64	The United States	cross-sectional study	Blood lead	Hypertension	NO	YES	NO	YES
Wen-Yi Yang (2018) (Asleep hypertension)	0.92	0.73	1.16	The United States	cross-sectional study	Blood lead	Hypertension	NO	YES	NO	YES
Wen-Yi Yang (2018) (White coat effect)	1.36	1.01	1.82	The United States	cross-sectional study	Blood lead	Hypertension	NO	YES	NO	YES
Hui Miao (2020) (Men)	1.062	1.036	1.088	The United States	cross-sectional study	Blood lead	Uncontrolled Hypertension	Yes	Yes	NO	Yes
Hui Miao (2020) (Women)	1.056	1.011	1.102	The United States	cross-sectional study	Blood lead	Uncontrolled Hypertension	Yes	Yes	NO	Yes
Katherine M. Johnson (2020)	0.76	0.39	1.5	The United States	cross-sectional study	Bone lead	Hypertension In Pregnancy	NO	NO	NO	NO
Su Zhen Wu (2021) (Severe Preeclampsia)	1.1	0.72	1.68	China	cohort study	Blood lead	Hypertension In Pregnancy	NO	NO	NO	NO
Su Zhen Wu (2021) (Mild Preeclampsia)	1.62	1.27	2.06	China	cohort study	Blood lead	Hypertension In Pregnancy	NO	NO	NO	NO
Hai Duc Nguyen (2021) (Premenopausal women)	1.27	0.39	4.12	South korea	cross-sectional study	Blood lead	hypertension during menopause	Yes	Yes	Yes	NO
Hai Duc Nguyen (2021) (Postmenopausal women)	1.16	0.63	2.15	South korea	cross-sectional study	Blood lead	hypertension during menopause	Yes	Yes	Yes	NO
Jeoung A. Kwon (2023) (Inactive)	1.7	1.34	2.16	South korea	cross-sectional study	Blood lead	Hypertension	Yes	Yes	NO	Yes
Jeoung A. Kwon (2023) (Moderate active)	1.5	1	2.25	South korea	cross-sectional study	Blood lead	Hypertension	Yes	Yes	NO	Yes
Jeoung A. Kwon (2023) (Most active)	2.24	1.19	4.23	South korea	cross-sectional study	Blood lead	Hypertension	Yes	Yes	NO	Yes
Hao Chen (2023) (Men RHTN vs. NRHTN)	1.18	0.95	1.46	The United States	cross-sectional study	Blood lead	Resistant Hypertension	Yes	Yes	NO	Yes
Hao Chen (2023) (Men RHTN vs. NHTN)	1.57	1.24	1.99	The United States	cross-sectional study	Blood lead	Resistant Hypertension	Yes	Yes	NO	Yes
Hao Chen (2023) (Men NRHTN vs. NHTN)	1.33	1.21	1.45	The United States	cross-sectional study	Blood lead	Hypertension	Yes	Yes	NO	Yes
Hao Chen (2023) (Women RHTN vs. NRHTN)	1.12	0.94	1.34	The United States	cross-sectional study	Blood lead	Resistant Hypertension	Yes	Yes	NO	Yes
Hao Chen (2023) (Women RHTN vs. NHTN)	1.81	1.49	2.19	The United States	cross-sectional study	Blood lead	Resistant Hypertension	Yes	Yes	NO	Yes
Hao Chen (2023) (Women NRHTN vs. NHTN)	1.61	1.45	1.79	The United States	cross-sectional study	Blood lead	Hypertension	Yes	Yes	NO	Yes
Hao Chen (2023) (≤60 years RHTN vs. NRHTN)	1.33	1.02	1.75	The United States	cross-sectional study	Blood lead	Resistant Hypertension	Yes	Yes	NO	Yes
Hao Chen (2023) (≤60 years RHTN vs. NHTN)	2.24	1.7	2.97	The United States	cross-sectional study	Blood lead	Resistant Hypertension	Yes	Yes	NO	Yes
Hao Chen (2023) (≤60 years NRHTN vs. NHTN)	1.68	1.53	1.85	The United States	cross-sectional study	Blood lead	Hypertension	Yes	Yes	NO	Yes
Hao Chen (2023) (>60 years RHTN vs. NRHTN)	1.13	0.98	1.3	The United States	cross-sectional study	Blood lead	Resistant Hypertension	Yes	Yes	NO	Yes
Hao Chen (2023) (>60 years RHTN vs. NHTN)	1.11	0.95	1.29	The United States	cross-sectional study	Blood lead	Resistant Hypertension	Yes	Yes	NO	Yes
Hao Chen (2023) (>60 years NRHTN vs. NHTN)	0.99	0.88	1.1	The United States	cross-sectional study	Blood lead	Hypertension	Yes	Yes	NO	Yes
Hao Chen (2023) (Kidneydysfunction RHTN vs. NRHTN)	1.28	1.08	1.5	The United States	cross-sectional study	Blood lead	Resistant Hypertension	Yes	Yes	NO	Yes
Hao Chen (2023) (Kidneydysfunction RHTN vs. NHTN)	1.74	1.43	2.11	The United States	cross-sectional study	Blood lead	Resistant Hypertension	Yes	Yes	NO	Yes
Hao Chen (2023) (Kidneydysfunction NRHTN vs. NHTN)	1.36	1.19	1.56	The United States	cross-sectional study	Blood lead	Hypertension	Yes	Yes	NO	Yes
Hao Chen (2023) (Nokidneydysfunction RHTN vs. NRHTN)	0.99	0.78	1.25	The United States	cross-sectional study	Blood lead	Resistant Hypertension	Yes	Yes	NO	Yes
Hao Chen (2023) (Nokidneydysfunction RHTN vs. NHTN)	1.48	1.17	1.88	The United States	cross-sectional study	Blood lead	Resistant Hypertension	Yes	Yes	NO	Yes
Hao Chen (2023) (Nokidneydysfunction NRHTN vs. NHTN)	1.5	1.37	1.63	The United States	cross-sectional study	Blood lead	Hypertension	Yes	Yes	NO	Yes

**Table 3:** Dose–response baseline table of blood lead level and hypertension incidence.

Author	*N*	Cases	Grouping basis	Blood lead level	OR	LCI	UCI
Tiange Liu (2019)	255	22	/	Q1 (0.058–0.156) μg/dl	1		
	258	22		Q2 (0.157–0.21) μg/dl	1.05	0.59	1.85
	254	26		Q3 (0.212–0.28) μg/dl	1.19	0.68	2.1
	253	23		Q4 (0.281–0.428) μg/dl	1.03	0.57	1.88
	254	22		Q5 (0.43–2.48) μg/dl	0.9	0.48	1.68
Min Gi Kim (2019)	1839	178	/	1st (0.01–3.68) μg/dl	1		
	1849	193		2nd (3.69–5.19) μg/dl	1.04	0.83	1.29
	1820	232		3th (5.20–6.86) μg/dl	1.2	0.97	1.48
	1833	299		4th (6.87–10.00) μg/dl	1.54	1.26	1.89
Hui Miao (2020)	2,838	1,205	Men	Q1 (<0.94) μg/dl	1		
	3,590	1850		Q2 (0.94–1.50) μg/dl	1.191	1.032	1.375
	4,055	2,253		Q3 (1.50–2.30) μg/dl	1.331	1.169	1.515
	5,196	3,230		Q4 (>2.30) μg/dl	1.48	1.28	1.71
	3,047	871	Women	Q1 (<0.70) μg/dl	1		
	3,544	1,468		Q2 (0.70–1.08) μg/dl	1.158	0.968	1.386
	3,974	2,115		Q3 (1.08–1.66) μg/dl	1.244	1.041	1.486
	4,518	2,859		Q4 (>1.66) μg/dl	1.316	1.08	1.603
Su Zhen Wu (2021)	498	10	/	Q1 (2.00–2.90) μg/dl	1		
	514	14		Q2 (3.00–3.60) μg/dl	1.48	0.64	3.39
	488	8		Q3 (3.70–4.40) μg/dl	0.85	0.33	2.2
	615	27		Q4 (4.50–7.90) μg/dl	2.38	1.13	5.03
Jeoung A. Kwon (2023)	1,217	154	Inactive	Q1 (<1.60) μg/dl	1		
	2,829	607		Q2 (1.60–2.81) μg/dl	1.25	0.96	1.62
	1,296	434		Q3 (≥2.81) μg/dl	1.75	1.29	2.37
	450	45	Moderate active	Q1 (<1.60) μg/dl	1		
	1,434	293		Q2 (1.60–2.81) μg/dl	1.57	1.02	2.4
	790	262		Q3 (≥2.81) μg/dl	2.2	1.36	3.58
	82	13	Most active	Q1 (<1.60) μg/dl	1		
	340	79		Q2 (1.60–2.81) μg/dl	1.26	0.54	2.94
	240	85		Q3 (≥2.81) μg/dl	2.08	0.88	4.92

The funnel plot showed marked asymmetry, with most small studies clustering on the side of larger effect sizes, suggesting possible publication bias or small-study effects. Subsequent Egger’s regression confirmed this finding: the intercept deviated significantly from zero (95% CI did not contain 0), indicating evident small-study effects or potential publication bias. Consequently, the pooled effect estimate may be inflated by publication bias. Sensitivity analyses revealed that sequentially omitting each individual study left the pooled effect estimate virtually unchanged (OR 1.27, 95% CI 1.19–1.37). The point estimate consistently hovered around 1.27, with the lower bound of the 95% confidence interval remaining above 1.19 and the upper bound below 1.37 for every leave-one-out iteration. These findings indicate that the overall results are not disproportionately influenced by any single study and demonstrate robust stability (Supplementary Figures 1–3).

The results showed that the quality score of case–control study was 7–9, the quality score of cohort study was 5–8, and the quality score of cross-sectional study was 5–9. The overall literature quality was in the middle and upper level. Details of NOS and JBI scores are attached (Supplementary Tables 8–10).

### 3.2 Subgroup analysis

This study employed meta-regression analysis to investigate the influence of potential factors on the heterogeneity of the pooled effect size. We adhered to a strict statistical significance threshold of *p* < 0.05, accounting for the potential for multiple comparisons. The analysis revealed that, among all covariates examined, only lead exposure emerged as a significant moderator of heterogeneity (*p* = 0.039). Although the *p*-value for alcohol consumption was 0.029 in univariate analysis, its association was not considered statistically significant after rigorous adjustment for multiple lifestyle covariates, including smoking, physical activity, and BMI. This suggests that the independent explanatory power of alcohol consumption for heterogeneity is limited when other co-variables are considered concurrently. Furthermore, other factors—including smoking (*p* = 0.190), physical activity level (*p* = 0.842), BMI (*p* = 0.876), hypertension type (*p* = 0.604), study design (*p* = 0.161), and country (*p* = 0.322)—were confirmed not to be significant sources of heterogeneity in this study. In summary, lead exposure was the only factor definitively identified to significantly explain the between-study heterogeneity in this meta-analysis (Supplementary Tables 4–11).

**Figure 3:**
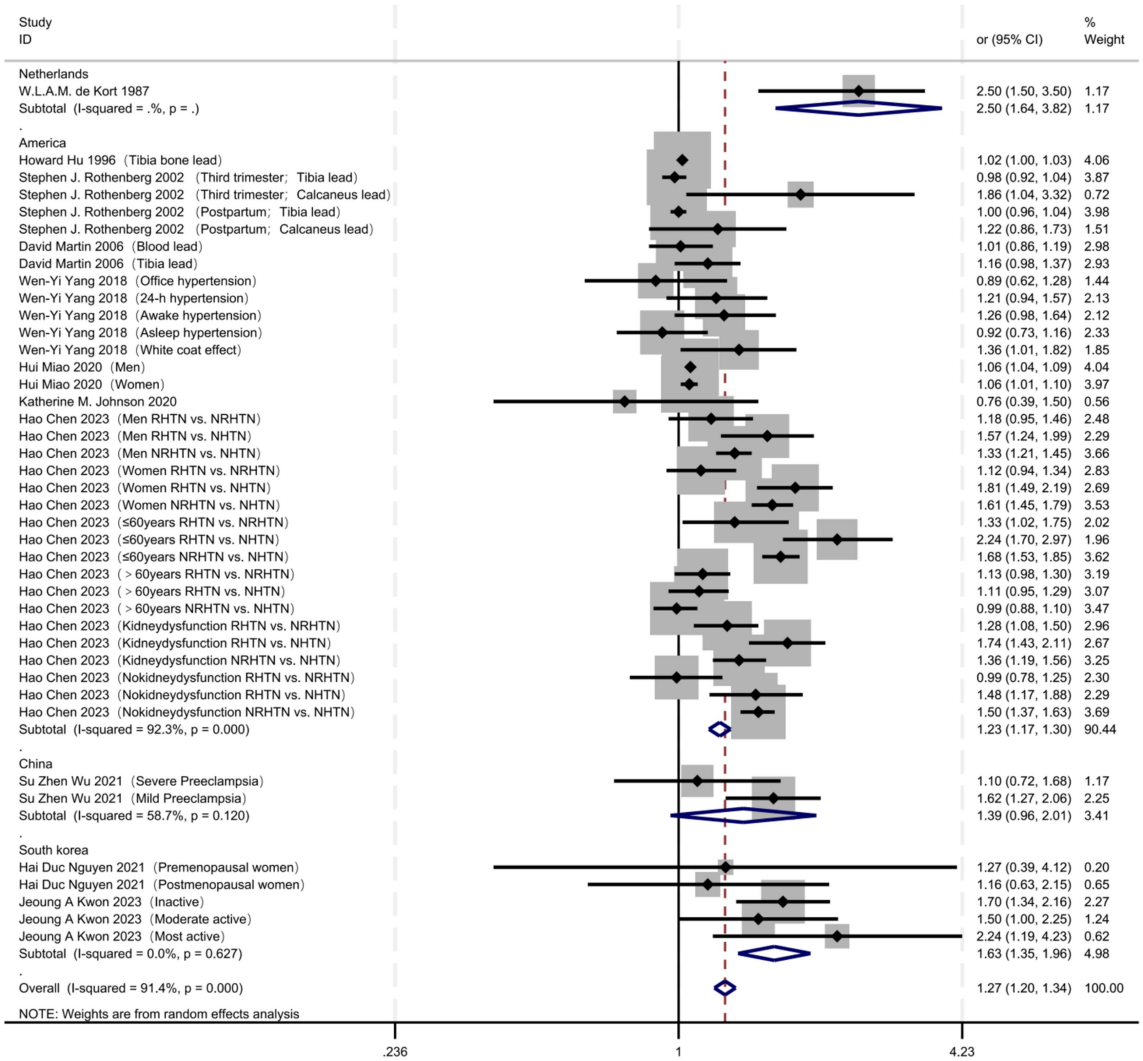
Regional differences: comparison of population association strength in different countries/regions.

Geographically (Figure 3; Table 4), studies from South Korea revealed a significant association between lead exposure and hypertension risk, with an OR of 1.63 (95% CI 1.35–1.96) and no heterogeneity across studies (I^2^ = 0.0%, *p* = 0.627). Investigations conducted in The United States indicated that lead exposure increases hypertension risk (OR = 1.23, 95% CI 1.17–1.30), although marked heterogeneity was present (I^2^ = 92.3%, *p* < 0.001). A single study from the Netherlands reported a strong association, yielding an OR of 2.50 (95% CI 1.64–3.82); heterogeneity analysis was not feasible. Studies performed in China did not observe a statistically significant relationship (OR = 1.39, 95% CI 0.96–2.01) and exhibited moderate heterogeneity (I^2^ = 58.7%, *p* = 0.120).

**Table 4:** Subgroup analysis summary of lead exposure and hypertension risk.

Subgroup analysis	Numbers of studies	Pooled results		Heterogeneity		Meta-regression
		OR (95% CI)	*p*	I^2^	*p*	
Study location						0.322
South Korea	5	**1.63 (1.35, 1.96)**	< 0.001	0.0%	0.627	
The United States	33	**1.23 (1.17, 1.30)**	< 0.001	92.3%	< 0.001	
Netherlands	1	**2.50 (1.64, 3.82)**	< 0.001	/	/	
China	2	1.39 (0.96, 2.01)	0.082	58.7%	0.120	
Type of research						0.161
Cross-sectional study	34	1.31 (1.21, 1.41)	< 0.001	90.0%	< 0.001	
Cohort study	6	1.11 (0.99, 1.25)	0.072	76.6%	0.120	
Case control study	1	1.02 (1.00, 1.03)	0.015	/	/	
Type of lead exposure						0.039
Blood lead	34	**1.32 (1.23, 1.42)**	< 0.001	90.2%	< 0.001	
Bone lead	7	1.01 (0.98, 1.05)	0.441	42.4%	0.108	
Types of hypertension						0.604
Hypertension during menopause	2	1.18 (0.69, 2.04)	0.546	0.0%	0.894	
Hypertension	18	**1.31 (1.16, 1.48)**	< 0.001	94.8%	< 0.001	
Resistant Hypertension	12	**1.36 (1.20, 1.55)**	< 0.001	79.9%	< 0.001	
Uncontrolled Hypertension	2	**1.06 (1.04, 1.08)**	< 0.001	0.0%	0.823	
Hypertension In Pregnancy	7	1.10 (0.98, 1.23)	0.100	72.7%	0.001	
Adjustment for drinking alcohol						0.029
Yes	26	**1.33 (1.24, 1.42)**	< 0.001	93.9%	< 0.001	
No	14	**1.10 (1.02, 1.19)**	0.018	91.4%	0.001	
Adjustment for smoking						0.190
Yes	34	**1.29 (1.20, 1.38)**	< 0.001	91.2%	< 0.001	
No	6	1.12 (0.97, 1.28)	0.111	70.9%	0.004	
Adjustment for physical activity						0.842
Yes	2	1.18 (0.69, 2.04)	0.546	0.0%	0.894	
No	38	**1.26 (1.19, 1.33)**	< 0.001	91.8%	< 0.001	
Adjustment for BMI						0.876
Yes	35	**1.25 (1.19, 1.32)**	< 0.001	92.2%	< 0.001	
No	5	1.25 (0.94, 1.65)	0.119	34.6%	0.191	

**Figure 4:**
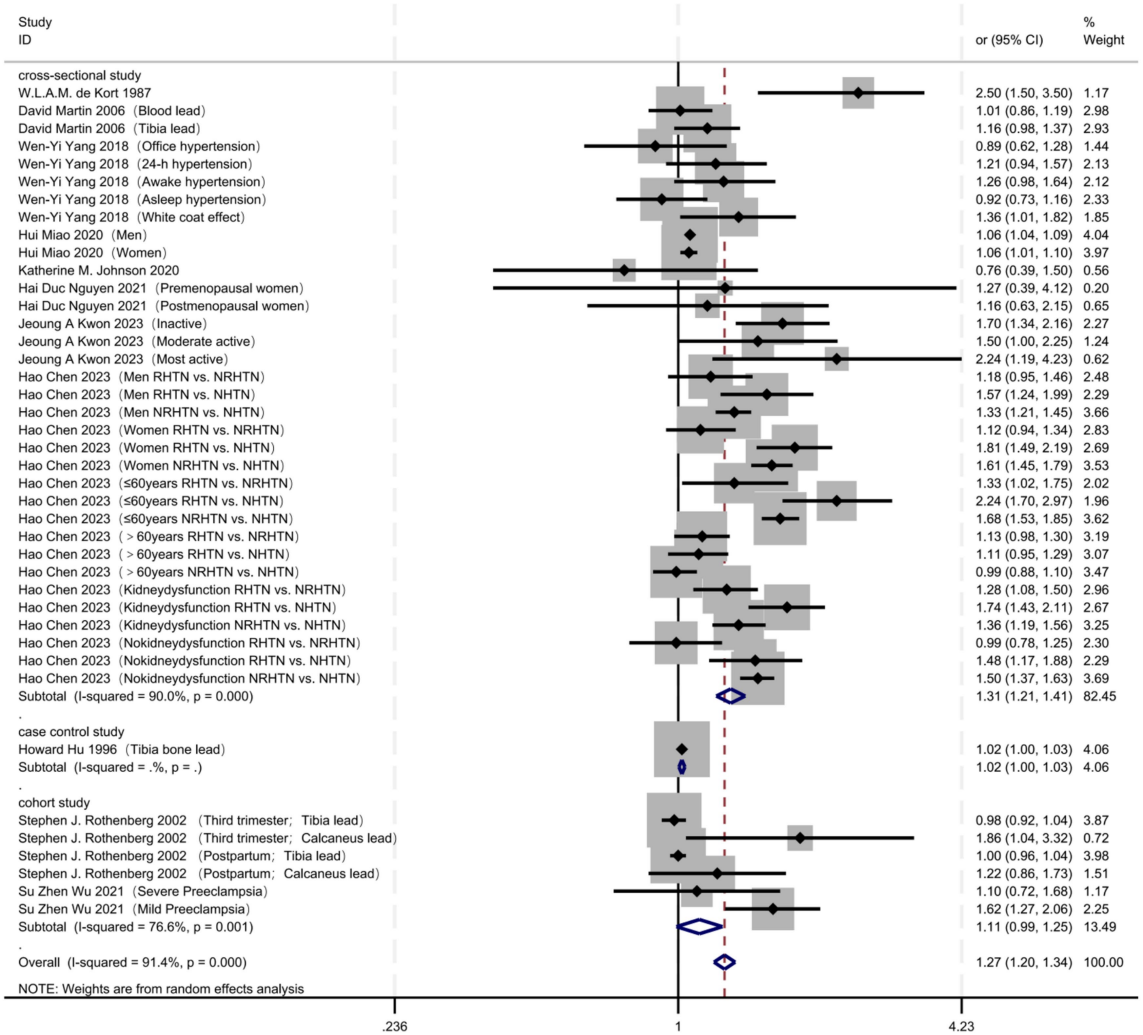
Study design: effect stratification of cross-sectional, cohort and case–control studies.

When stratified by study design (Figure 4; Table 4), cross-sectional studies revealed a significant association between lead exposure and hypertension (OR = 1.31, 95% CI 1.21–1.41) but exhibited extreme heterogeneity (I^2^ = 90.0%, *p* < 0.001). Cohort studies did not detect a statistically significant relationship (OR = 1.11, 95% CI 0.99–1.25) and displayed moderate heterogeneity (I^2^ = 76.6%, *p* = 0.001). Case–control studies indicated only a marginal association (OR = 1.02, 95% CI 1.00–1.03), with no heterogeneity statistics available.

**Figure 5:**
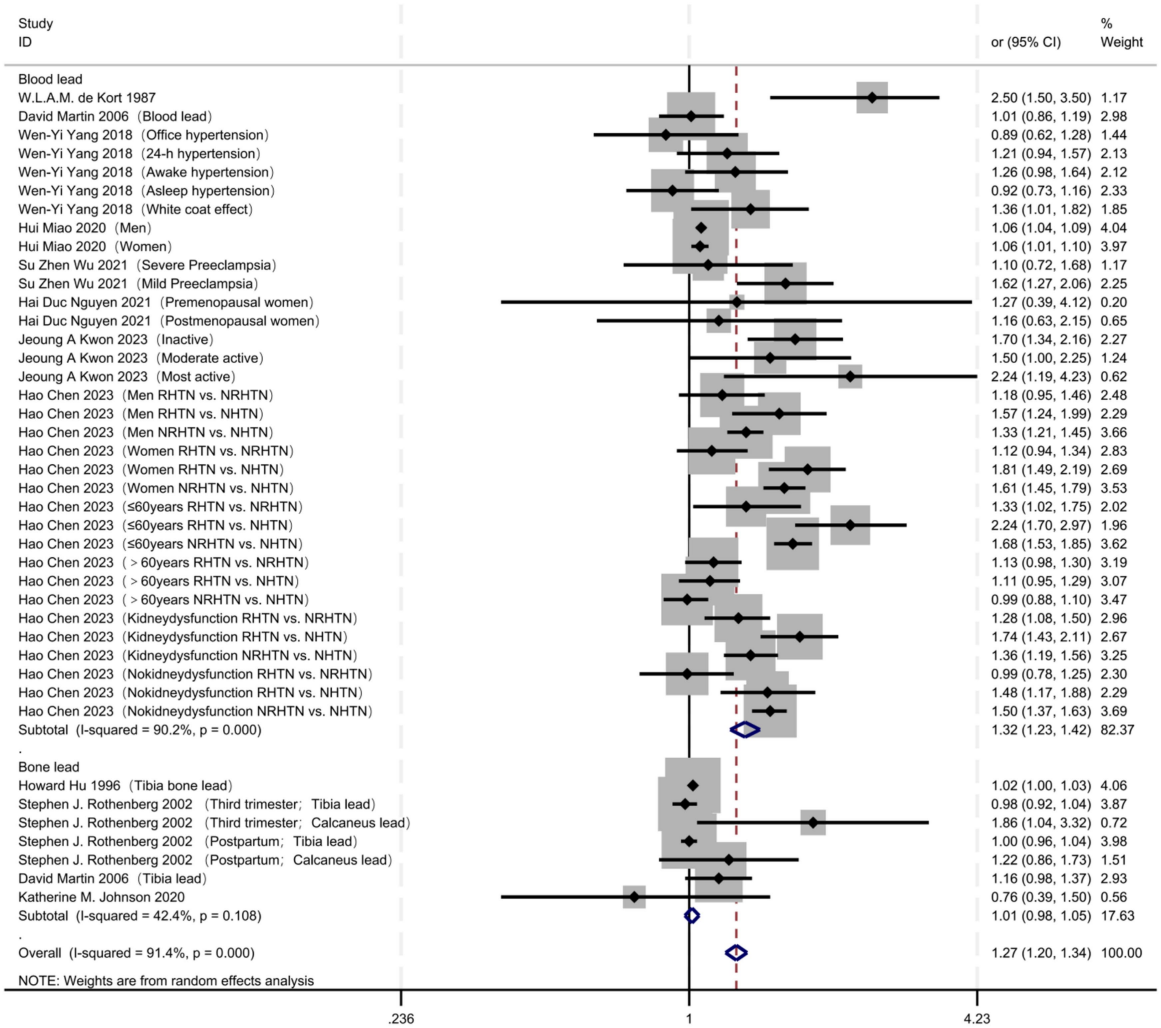
Biomarkers: comparison of the effects of blood lead and bone lead on hypertension.

With regard to exposure type (Figure 5; Table 4), blood lead exposure was significantly associated with hypertension (OR = 1.32, 95% CI 1.23–1.42), although heterogeneity was extreme (I^2^ = 90.2%, *p* < 0.001). Conversely, bone lead exposure showed no significant association (OR = 1.01, 95% CI 0.98–1.05) and exhibited moderate heterogeneity (I^2^ = 42.4%, *p* = 0.108).

**Figure 6:**
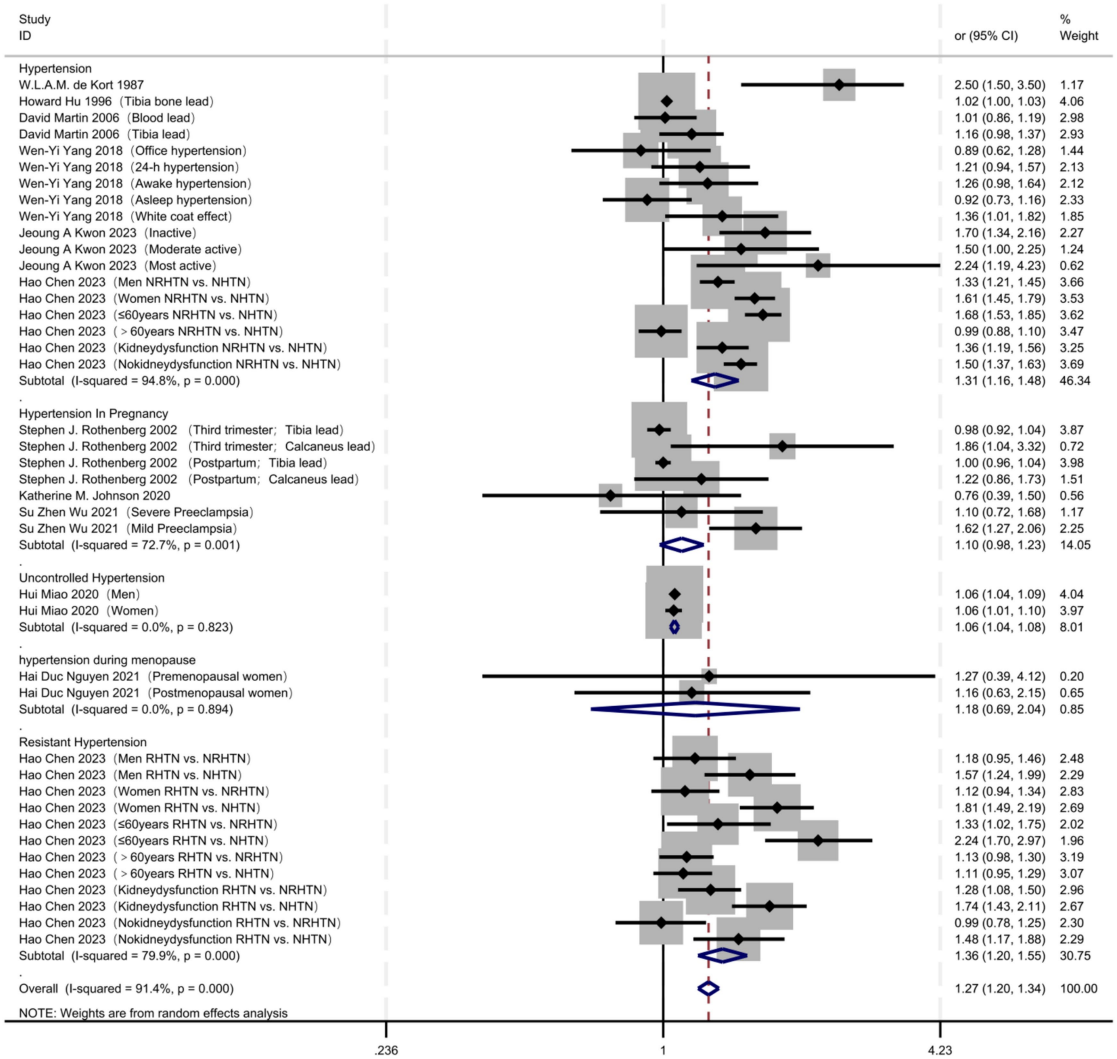
Subtypes of hypertension: differences in the association between common, refractory, pregnancy and uncontrolled hypertension.

Regarding hypertension subtypes (Figure 6; Table 4), lead exposure significantly increased the risk of essential hypertension (OR = 1.31, 95% CI 1.16–1.48), with extreme heterogeneity (I^2^ = 94.8%, *p* < 0.001). A significant association was also observed for resistant hypertension (OR = 1.36, 95% CI 1.20–1.55), accompanied by high heterogeneity (I^2^ = 79.9%, *p* < 0.001). For uncontrolled hypertension, a modest yet significant association was found (OR = 1.06, 95% CI 1.04–1.08) and no between-study heterogeneity was detected (I^2^ = 0.0%, *p* = 0.823). No significant relationship was seen with gestational hypertension (OR = 1.10, 95% CI 0.98–1.23) and substantial heterogeneity was present (I^2^ = 72.7%, *p* = 0.001). Similarly, menopausal hypertension exhibited no significant association (OR = 1.18, 95% CI 0.69–2.04) and no inter-study heterogeneity (I^2^ = 0.0%, *p* = 0.894).

In terms of confounder adjustment (Supplementary Figures 12–15; Table 4), the association became more pronounced after adjustment for alcohol consumption (OR = 1.33, 95% CI 1.24–1.42). After controlling for smoking, the association remained significant (OR = 1.29, 95% CI 1.20–1.38) but with high heterogeneity. Adjustment for BMI also yielded a significant association (OR = 1.25, 95% CI 1.19–1.32), accompanied by considerable heterogeneity. In contrast, adjustment for physical activity resulted in a non-significant association (OR = 1.18, 95% CI 0.69–2.04) and no inter-study heterogeneity (I^2^ = 0.0%, *p* = 0.894).

### 3.3 Dose–response analysis

**Figure 7:**
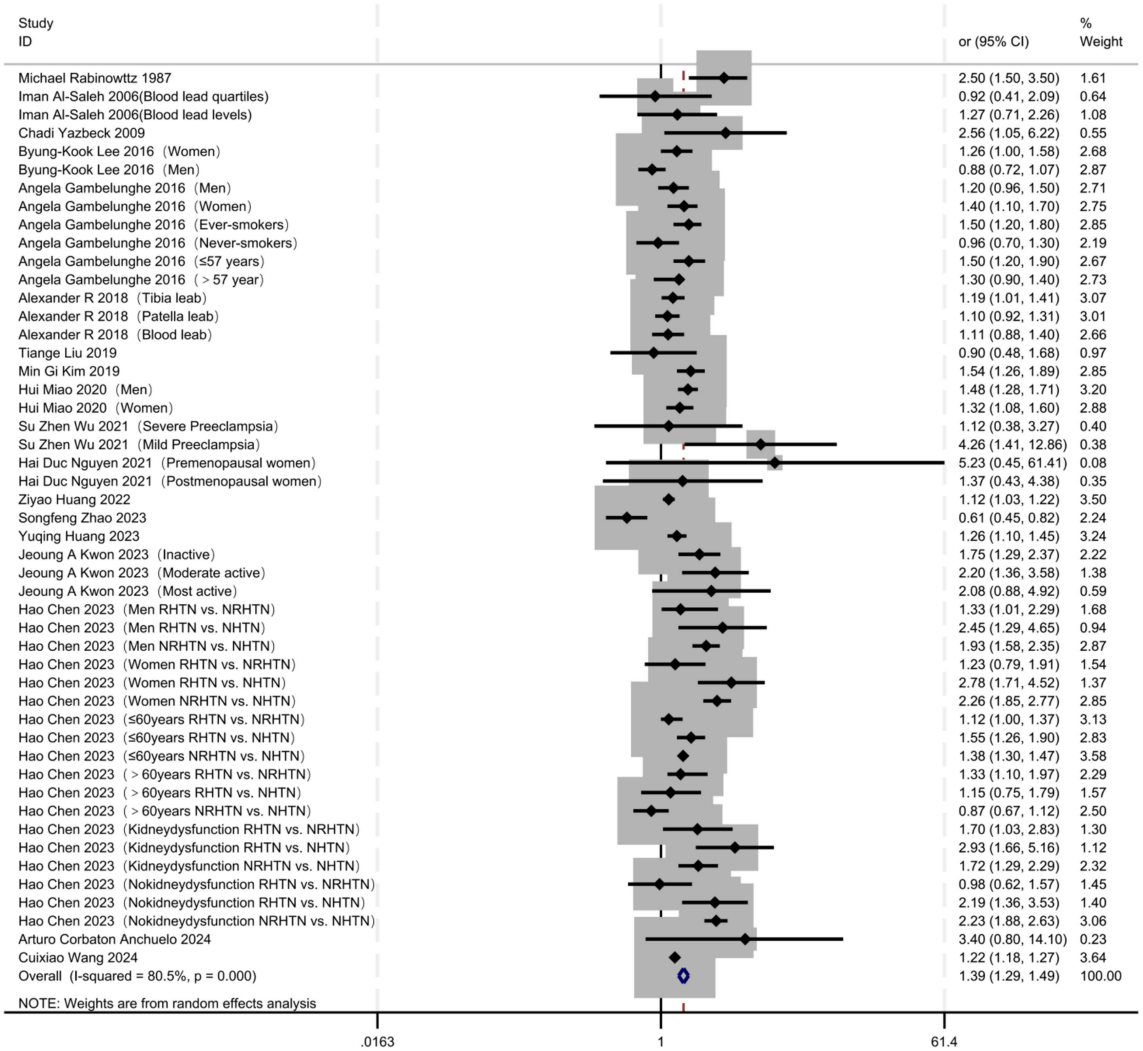
Extreme exposure: summary of the highest vs. the lowest lead exposure levels for hypertension risk.

**Figure 8:**
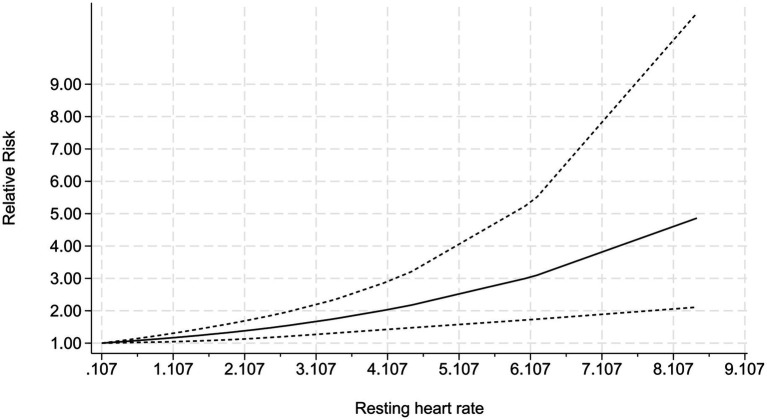
Dose–response: non-linear relationship between continuous increase of blood lead and the risk of hypertension.

An analysis of 18 studies that reported ORs for the highest quantile of lead exposure demonstrated a significantly elevated risk of hypertension in the highest versus the lowest exposure category, with a pooled OR of 1.39 (95% CI 1.29–1.49) and marked between-study heterogeneity (I^2^ = 80.5%, *p* < 0.001) (Figure 7). Collectively, the findings confirm a dose–response relationship in which increasing lead exposure is accompanied by progressively higher hypertension risk, providing quantitative evidence that lead exposure is a risk factor for hypertension and supporting the inference of a causal association (Figure 8). The funnel plot exhibited pronounced asymmetry, with small studies clustered toward larger effect sizes, and Egger’s regression intercept deviated significantly from zero (95% CI did not contain 0), indicating potential publication bias or small-study effects that may inflate the pooled estimate. Nevertheless, sensitivity analyses showed that the pooled effect remained stable within a narrow range (OR ≈ 1.27–1.52) after sequentially excluding any single study, with the lower bound of the 95% CI consistently exceeding 1.27 and no substantial fluctuation, underscoring the robustness of the overall results (Supplementary Figures 16–18).

Five publications, contributing eight data points, reported blood lead levels together with incident hypertension cases; among them, Hui Miao presented sex-specific strata (men and women), while Jeoung A Kwon stratified by physical activity (inactive, moderate active, most active). Dose–response analysis revealed an overall upward trend in hypertension risk with increasing blood lead levels. When blood lead remained low (e.g., ≤ 0.107 μg/dl), the odds ratio approached 1.00, indicating a weak association with hypertension. At a blood lead concentration of 8.435 μg/dl, the odds ratio rose sharply to approximately 4.85, denoting a markedly elevated risk. Notably, once blood lead exceeded 6.14 μg/dl, the incidence of hypertension accelerated rapidly (Supplementary Table 7). Specifically, at 1.107 μg/dl the OR was 1.16 (95% CI: 1.31–1.04), at 3.107 μg/dl it reached 1.65 (95% CI: 2.18–1.26), and at 6.107 μg/dl it further increased to 3.03 (95% CI: 5.35–1.72). These findings provide clear evidence of a positive dose–response relationship: higher blood lead concentrations are accompanied by progressively greater hypertension risk.

## 4 Discussion

### 4.1 Main findings and heterogeneity sources

This study presents the first comprehensive meta-analysis to evaluate the association between environmental and occupational lead exposure and various hypertension subtypes, while also clarifying the dose–response relationship. Our findings show that lead exposure significantly increases the risk of developing hypertension (pooled OR = 1.27, 95% CI: 1.20–1.34), with notable heterogeneity across exposure levels, hypertension phenotypes, and population characteristics. Meta-regression analyses revealed that the method of lead exposure assessment—blood lead vs. bone lead—was a major source of between-study variability (*p* = 0.039). Multivariable adjustment for key lifestyle and metabolic factors significantly modulated the pooled effect estimates.

Importantly, blood lead does not merely indicate short-term exposure but also reflects ongoing mobilization of lead from bone, particularly under conditions of increased bone resorption such as pregnancy, menopause, or aging. These dynamic exchanges between compartments may underlie the stronger and more variable associations observed for blood lead compared to bone lead. Multivariable adjustment for key lifestyle and metabolic factors significantly modulated the pooled effect estimates. Notably, adjustment for physical activity completely attenuated the association, suggesting that its effect may be largely mediated or confounded by other factors included in the model. Dose–response modeling demonstrated a monotonic increase in hypertension risk with rising blood lead levels, consistent with both exogenous and endogenous contributions to circulating lead burden.

Below 0.107 μg/dl, the association was weak (OR ≈ 1.0), but beyond this threshold, the risk rose sharply, reaching an OR of 4.85 (95% CI: 2.11–11.09) at 8.435 μg/dl. These findings not only support the biological plausibility of lead as a risk factor for hypertension but also highlight 0.107 μg/dl as a potential action threshold for public health intervention, offering a quantitative basis for primary prevention strategies. Notably, while major international guidelines (e.g., WHO/CDC) still use 5 μg/dl as the intervention cutoff, a growing body of epidemiological evidence consistently shows that cardiovascular risk begins to climb at much lower lead exposure levels—often below 2–3 μg/dl—and follows a no-threshold dose–response pattern (18, 19).

### 4.2 Context within environmental metal mixtures and cardiovascular risk

While this study focuses specifically on lead exposure, it is important to view the associated risks within the broader context of environmental metal mixtures. A growing body of evidence suggests that multiple toxic metals can harm the cardiovascular system, acting either independently or synergistically. Two major systematic reviews have consistently shown that toxic metal pollutants significantly increase the risk of cardiovascular disease and stroke (20, 21).

A 2018 BMJ review—including 37 studies and approximately 348,000 participants—found a linear dose–response relationship between blood lead levels and coronary heart disease (RR 1.07 per 5 μg/dl increase; 95% CI: 1.04–1.10) (20). In addition to lead, arsenic, cadmium, and copper were also associated with an increased risk of cardiovascular events, while mercury showed no significant association. Similarly, a 2022 meta-analysis on stroke—pooling data from 38 studies and over 640,000 individuals—reported that high exposure to lead, cadmium, and copper increased stroke risk by 7% (1.07; 1.00–1.14), 30% (1.30; 1.13–1.48), and 19% (1.19; 1.04–1.36), respectively (21). Arsenic and mercury were not significantly associated with stroke in this analysis. Both studies highlight that cardiovascular risks rise with increasing exposure levels, reinforcing the clinical relevance of even current low-level exposures. These findings support the inclusion of blood lead and cadmium levels in cardiovascular risk assessments and provide a strong rationale for establishing evidence-based public health intervention thresholds. Notably, hypertension is a key intermediate pathway linking toxic metal exposure to cardiovascular disease and stroke. Substantial evidence supports this mechanistic connection and also reveals distinct exposure–response patterns across metals. Cadmium exposure, for instance, is positively associated with hypertension risk. Urinary cadmium shows a near-linear relationship with risk, while blood cadmium exhibits a steep increase at low concentrations (< 2 μg/L) before plateauing (22, 23). Mercury exposure also correlates with higher blood pressure and hypertension, with a hair mercury level of 3 g/g identified as a toxicological tipping point (24). In populations with elevated mercury levels (hair mercury ≥2 μg/g), the risk of hypertension is particularly pronounced. Chronic arsenic exposure has similarly been linked to increased hypertension risk, especially elevated systolic blood pressure, with evidence of a nonlinear dose–response relationship (25).

Lead (Pb) exposure is one of the most extensively studied toxic metals, and its effect on blood pressure is particularly well-established. While recent prospective studies are relatively scarce and often rely on data dating back one to two decades, multiple high-quality meta-analyses have consistently shown two key findings: (1) A doubling of blood lead levels is associated with a 1.0 mmHg increase in systolic and 0.6 mmHg in diastolic blood pressure, suggesting that even low-level exposure continues to elevate population-level blood pressure. (2) Tibial bone lead, a marker of long-term internal exposure, shows that every 10 μg/g increase is linked to a 4% higher risk of hypertension, with a cumulative effect on systolic BP of approximately 0.26 mmHg (26, 27). These findings are based on two large meta-analyses which collectively included more than 58,000 individuals from both the general population and occupationally exposed groups. The results remained consistent across sexes and after adjusting for a wide range of confounders, confirming that lead can raise blood pressure and amplify hypertension risk through both recent (blood lead) and long-term (bone lead) exposure pathways. Therefore, lead exposure should be considered a modifiable and significant public health risk factor for hypertension. Importantly, however, most previous studies did not systematically differentiate the dynamic roles of different biomarkers (e.g., blood vs. bone lead), nor did they deeply explore specific hypertension subtypes. To address this gap, our study goes beyond simply confirming lead’s harmful effects. For the first time, we used dose–response meta-analysis to quantify the exposure threshold for blood lead and hypertension risk, identifying 0.107 μg/dl as a key inflection point. We also found that risk rises sharply above 6.14 μg/dl, highlighting a non-linear exposure–response relationship. Even more notably, our subgroup analyses revealed a significant link between blood lead and resistant hypertension (OR = 1.36), while the association with bone lead did not reach statistical significance. This suggests that blood lead may be a more sensitive and clinically useful marker for early screening and intervention. In summary, lead exposure is a clear, modifiable risk factor for hypertension. Like other toxic metals such as cadmium and arsenic, reducing environmental lead exposure should be a core target for primary cardiovascular prevention.

### 4.3 Distinct roles of blood lead versus bone lead

One of the clearest conclusions from our meta-analysis is that elevated blood lead concentration is strongly associated with an increased risk of hypertension (pooled OR = 1.32, 95% CI: 1.23–1.42, I^2^ = 90%), whereas bone lead shows no significant association (OR = 1.01, 95% CI: 0.98–1.05, I^2^ = 42%). These findings from our meta-analysis provide a broad overview of the association between lead exposure and hypertension risk, but they do not fully capture the physiological complexity underlying blood lead levels in specific populations. In particular, variations in bone lead mobilization, influenced by factors such as pregnancy, lactation, and menopause, may modulate circulating blood lead concentrations and thus partly explain differences in hypertension risk across subgroups.

Increases in blood lead among pregnant and postmenopausal women are closely related to bone lead mobilization and have been linked to elevated blood pressure. This topic has been supported and expanded by multiple studies. Bone serves as the primary endogenous reservoir of lead, and its contribution to circulating blood lead becomes especially pronounced during periods of accelerated bone turnover, such as pregnancy and postmenopausal osteoporosis. In perimenopausal women, blood lead levels are among the highest observed across study populations (28), reflecting enhanced skeletal lead release. Pregnancy also accelerates bone turnover, leading to increased blood lead levels; this elevation is particularly evident in the second trimester and correlates with markers of bone resorption (29).

These observations are further supported by recent research. Korrick et al. demonstrated that tibia and patella lead levels were significantly associated with blood lead only in postmenopausal women not using estrogen, indicating that estrogen deficiency–related bone resorption mobilizes skeletal lead into the circulation (30). Gulson et al. showed that bone-derived lead contributed approximately 50% of circulating blood lead in late pregnancy, increasing to 70–90% during lactation, confirming that endogenous bone release is a major determinant of blood lead in this period (31). Additionally, Tong et al. reported a positive association between blood lead and FSH in postmenopausal women, suggesting that bone turnover mediates blood lead variation even in low-exposure populations, whereas blood lead itself was not directly associated with bone mineral density or fracture risk (32).

Collectively, these findings highlight that blood lead levels reflect not only recent environmental exposure but also dynamic redistribution from bone and other tissues. Incorporating this multi-compartment perspective is crucial for understanding the mechanisms underlying blood lead–associated blood pressure elevations, particularly in women experiencing accelerated bone turnover during pregnancy and menopause.

### 4.4 Subgroup analyses: hypertension phenotypes and study characteristics

Subgroup analysis showed that there were significant differences in the association between lead exposure and different hypertension subtypes. Primary hypertension (OR = 1.31, 95% CI: 1.16 ~ 1.48) and resistant hypertension (OR = 1.36, 95% CI: 1.20 ~ 1.55) were strongly positively correlated with lead exposure, while gestational hypertension (OR = 1.10, 95% CI: 0.98 ~ 1.23) and menopausal hypertension (OR = 1.18, 95% CI: 0.69 ~ 2.04) did not reach statistical significance. These differences may be due to the subtype-specific pathophysiological mechanism and the regulatory effect of hormones on lead toxicity. Refractory hypertension is defined as the situation where blood pressure cannot be controlled even if five or more different types of antihypertensive drugs are used. This type of hypertension is closely related to the enhancement of sympathetic nerve activity and may be particularly sensitive to lead exposure. Lead exposure can lead to up-regulation of renal angiotensin II (Ang II) expression and enhance sympathetic nerve activity, thereby exacerbating the condition of hypertension (33, 34). The link between lead exposure and hypertension is that lead can increase the level of Ang II by increasing the activity of angiotensin converting enzyme (ACE), which in turn leads to increased blood pressure (35). Pregnancy-induced hypertension (PIH) is one of the common complications during pregnancy, and its pathological mechanism is complex and diverse. Among them, elevated estrogen levels and placental isolation of lead may be considered key factors. Studies have shown that elevated estrogen levels can fight against the occurrence of gestational hypertension through a variety of mechanisms. For example, estrogen can improve vascular function by inhibiting oxidative stress and inflammatory response. It can also protect the placenta from hypoxia-reoxidation damage by activating G protein-coupled receptor 30 (GPR30), reducing apoptosis and increasing cell proliferation, thereby improving placental perfusion (36). Together, these mechanisms may help to reduce the risk of gestational hypertension. In addition, the isolation of placenta from lead is also an important protective mechanism. Studies have found that the placenta during pregnancy can effectively isolate lead in the environment, thereby reducing the toxic effects of lead on the mother and fetus (8). In addition, changes in the activity of some enzymes in the placenta may also be related to the isolation of lead. For example, enhanced function of 11β-hydroxysteroid dehydrogenase 2 (11β-HSD2) reduces lead-induced calmodulin activation, thereby lowering blood pressure (37). For menopausal hypertension, the association was not statistically significant may be due to the small sample size (only 2 studies), or the effect of estrogen deficiency on vascular stiffness masked the relatively mild effect of lead (38). These subtype-specific patterns emphasize the need for individualized risk assessment. For example, blood lead monitoring should be prioritized for patients with resistant hypertension.

Study design was an important moderator of the effect size: the association strength observed in the cross-sectional study (OR = 1.31, 95% CI: 1.21–1.41, I^2^ = 90.0%) was higher than that in the cohort study (OR = 1.11, 95% CI: 0.99–1.25, I^2^ = 76.6%). This difference may be due to reverse causality in cross-sectional studies (e.g., undiagnosed hypertension alters lead metabolism) or effect dilution due to long-term follow-up in cohort studies (e.g., lead exposure or lifestyle changes over time) (39–41). Geographically, the OR values of studies in South Korea (OR = 1.63, 95% CI: 1.35 ~ 1.96, I^2^ = 0.0%) and the Netherlands (OR = 2.50, 95% CI: 1.64 ~ 3.82) were higher than those in the United States (OR = 1.23, 95% CI: 1.17 ~ 1.30) or China (OR = 1.39, 95% CI: 0.96 ~ 2.01). Meta-regression excluded the possibility of ‘country ‘as a significant source of heterogeneity (*p* = 0.322), suggesting that these differences may be due to unmeasured confounding factors, such as population-level calcium intake (which can reduce lead absorption) (42, 43). It is worth noting that no regional study results dominated the combined effect, which confirmed the global correlation of lead as a risk factor for hypertension.

### 4.5 Influence of confounding and effect modification

Adjusting for lifestyle and metabolic-related confounding factors highlighted their complex influence on the association between lead and hypertension. After adjusting for alcohol consumption (OR = 1.33, 95% CI: 1.24–1.42) or smoking (OR = 1.29, 95% CI: 1.20–1.38), the association remained robust, indicating that the effect of lead exposure on hypertension was independent of these risk factors. In contrast, after adjusting for physical activity, the association was attenuated to statistical non-significance (OR = 1.18, 95% CI: 0.69–2.04). This may be because regular physical activity can enhance the body’s antioxidant defense capacity (e.g., by increasing superoxide dismutase activity), thereby offsetting lead-induced oxidative stress (44, 45). After adjusting for body mass index (BMI), the effect size was slightly reduced (OR = 1.25, 95% CI: 1.19–1.32). The strength of the well-known association between BMI and hypertension can vary across different demographic groups (46). Furthermore, research has revealed differential effects of BMI on the lead-hypertension relationship across racial and gender groups. For instance, in a study of non-Hispanic White and non-Hispanic Black individuals, the positive association between blood lead levels and both systolic and diastolic blood pressure remained significant after BMI adjustment, whereas the association was not statistically significant among Hispanic and other racial groups (47). Thus, it is crucial to consider the influence of BMI when studying the relationship between lead and hypertension. These findings emphasize the importance of comprehensive confounder control in observational studies and suggest that public health interventions targeting lead exposure may be particularly effective in sedentary or obese individuals, as their intrinsic protective mechanisms may be limited.

### 4.6 Dose–response relationship and public health implications

Dose–response analysis revealed a monotonically increasing trend in hypertension risk as blood lead levels increased, identifying two key thresholds (1): an effect threshold of approximately 0.107 μg/dl, below which the OR was close to 1.0 (indicating minimal risk); and (2) an acceleration threshold of approximately 6.14 μg/dl, after which the risk increased sharply. The 0.107 μg/dl threshold may be attributable to the inhibition of eNOS and production of ROS caused by low blood lead concentrations (48–50), while the accelerated risk at 6.14 μg/dl may reflect the synergistic activation of multiple pathways (such as Ang II upregulation and sympathetic overexcitation) (48, 51). This finding contrasts with the current lead action threshold of 5 μg/dl for adults recommended by the U. S. Centers for Disease Control and Prevention (CDC). Our data indicate that this threshold may be insufficiently protective, as blood lead levels below 5 μg/dl are still associated with a measurable risk of hypertension. Our findings suggest that the blood lead action threshold for high-risk groups (such as patients with refractory hypertension or nephropathy) should be revised to < 0.107 μg/dl, and that blood lead detection should be included in routine hypertension risk assessment. Such evidence may help reduce the burden of hypertensive disease at the population level, especially in areas where environmental lead pollution persists (52).

## 5 Limitations and future directions

Despite the robust findings of this meta-analysis, several limitations should be acknowledged when interpreting the results. High Heterogeneity and Variable Study Quality: The overall analysis exhibited substantial heterogeneity (I^2^ = 91.4%), which, although partially explored through subgroup analyses, remains largely unexplained. This heterogeneity may stem from variations in study design, population characteristics, lead exposure assessment methods (e.g., blood lead vs. bone lead), and differences in adjustment for confounding factors across studies. The inclusion of studies with varying methodological quality may also influence the pooled estimate. Potential for Publication Bias: The funnel plot asymmetry and significant Egger’s test indicate a potential presence of publication bias or small-study effects, which may have inflated the overall effect size. Although sensitivity analysis confirmed the stability of the results, the possibility that smaller studies with null results were not published cannot be ruled out, potentially leading to an overestimation of the true effect. Limited Data in Specific Subgroups: Certain subgroups, such as studies from specific geographic regions (e.g., only one study from the Netherlands), particular hypertension subtypes (e.g., gestational and menopausal hypertension), and studies using bone lead as the exposure biomarker, were underrepresented. This limited the statistical power to draw firm conclusions for these strata.

Despite the above limitations, the results of this study still have clear public health significance. Future studies should focus on sensitive populations such as children, pregnant women, and older adults, and carry out multi-center, long-term follow-up prospective cohort studies, combined with dynamic monitoring of blood lead and individualized intervention to verify the findings of this study and explore the interaction between lead exposure and traditional risk factors. This study suggests that 0.107 μg/dl should be used as the public health action level for hypertension risk screening in the general population, and < 0.107 μg/dl should be used as the blood lead control target for high-risk groups (such as pregnant women and hypertensive patients).

## 6 Conclusion

In summary, this systematic review and meta-analysis confirms a significant association between lead exposure and incident hypertension, accompanied by a clear dose–response relationship that is modulated by geographic region, study design, exposure type, hypertension subtype, and confounding factors. These findings provide a scientific basis for incorporating lead exposure into the assessment framework for hypertension risk factors and support efforts to reduce population hypertension burden through control of environmental lead pollution. Nevertheless, several limitations exist, including small sample sizes in certain subgroups and substantial heterogeneity, so future high-quality cohort studies—combined with individual-level lead exposure monitoring and mechanistic investigations—are needed to further establish causality and identify potential intervention targets.

## Data Availability

The original contributions presented in the study are included in the article/Supplementary material, further inquiries can be directed to the corresponding authors.
