# Supplementary material for: Relationship between lead exposure and different types of hypertension: systematic review and dose–response meta-analysis
Source: Front Public Health. 2025 Dec 10;13:1706805. doi: 10.3389/fpubh.2025.1706805 (PMC12727891; doi:10.3389/fpubh.2025.1706805)
Supplement: Supplementary file 1 [file Data_Sheet_1.docx]

**Relationship between lead exposure and different types of hypertension : systematic review and dose-response meta-analysis**

**Supplementary Information**

Supplementary Table 1 Retrieval of PubMed.

Supplementary Table 2 Retrieval of Web of Science.

Supplementary Table 3 Retrieval of Embase.

Supplementary Table 4 Retrieval of EBSCO.

Supplementary Table 5 Retrieval of Scopus.

Supplementary Table 6 The OR value of the highest quantile of lead exposure.

Supplementary Table 7 Dose-response relationship between different blood lead levels and the risk of hypertension.

Supplementary Table 8 NOS quality score included in the case control study.

Supplementary Table 9 NOS quality score included in the cohort study.

Supplementary Table 10 JBI scale score included in cross-sectional studies.

Supplementary Table 11 PRISMA 2020 statement.

Supplementary Figure 1 Correlation sensitivity analysis.

Supplementary Figure 2 Correlation funnel plot.

Supplementary Figure 3 Correlation publication bias.

Supplementary Figure 4 Meta-regression based on country.

Supplementary Figure 5 Meta-regression based on study design.

Supplementary Figure 6 Meta-regression based on lead exposure measurement.

Supplementary Figure 7 Meta-regression based on hypertension subtype.

Supplementary Figure 8 Meta-regression based on drinking adjustment.

Supplementary Figure 9 Meta-regression based on smoking adjustment.

Supplementary Figure 10 Meta-regression based on physical-activity adjustment.

Supplementary Figure 11 Meta-regression based on BMI adjustment.

Supplementary Figure 12 Subgroup analysis based on drinking adjustment (adjusted vs. unadjusted).

Supplementary Figure 13 Subgroup analysis based on smoking adjustment (adjusted vs. unadjusted).

Supplementary Figure 14 Subgroup analysis based on physical-activity adjustment (adjusted vs. unadjusted).

Supplementary Figure 15 Subgroup analysis based on BMI adjustment (adjusted vs. unadjusted).

Supplementary Figure 16 The highest measurement sensitivity analysis.

Supplementary Figure 17 The highest dose funnel plot.

Supplementary Figure 18 Maximum dose publication bias.

**Supplementary table1 Retrieval of PubMed.**

| **Search via PubMed** | **Result** |
| --- | --- |
| #1 ((("Lead"[Mesh]) OR "Lead Poisoning"[Mesh]) OR "Metals, Heavy"[Mesh]) OR "Heavy Metal Poisoning"[Mesh] | 544 |
| #2 "Blood lead"[Title/Abstract] OR "Urinary lead"OR "Bone lead"[Title/Abstract] OR "Patella Lead "[Title/Abstract] OR ''Tibial lead''[Title/Abstract] OR ''Calcaneus lead''[Title/Abstract] |  |
| #3 #1 OR #2 |  |
| #4 "Essential Hypertension"[Mesh] OR "Hypertension, Pregnancy-Induced"[Mesh] OR "Hypertension"[Mesh] |  |
| #5 (''blood pressure''[Title/Abstract] OR ''Resistant Hypertension''[Title/Abstract] OR ''Gestational Hypertension''[Title/Abstract] OR ''Postpartum Hypertension''[Title/Abstract]) OR (''Secondary Hypertension''[Title/Abstract] OR ''hypertens*''[Title/Abstract] OR ''elevat* adj3 blood pressur*''[Title/Abstract] OR ''high adj3 blood pressur*''[Title/Abstract]) |  |
| #6 #4 OR #5 |  |
| #7 (Observational Study[Publication Type]) OR (''Cross-sectional''[Title/Abstract] OR ''Cohort''[Title/Abstract] OR ''Case-control study ''[Title/Abstract] OR ''retrospective study''[Title/Abstract] OR ''prospective study''[Title/Abstract]) |  |
| #8 ''relative risk'' OR ''RR'' OR ''hazard ratio'' OR ''HR'' OR ''odds ratio'' OR ''OR'' |  |
| #9 (#1 OR #2) AND (#4 OR #5) AND #7 AND #8 |  |

| **Supplementary table2 Retrieval of [Web of Science](https://www.webofscience.com/wos/?Init=Yes&SrcApp=CR&app=wos&locale=zh-CN).**   \| Search via [Web of Science](https://www.webofscience.com/wos/?Init=Yes&SrcApp=CR&app=wos&locale=zh-CN) \| Result \| \| --- \| --- \| \| #1 TS=(''lead'' OR ''lead poisoning'' OR '''heavy metals'' OR ''Heavy Metal Poisoning'' OR "Blood lead" OR "Urinary lead"OR "Bone lead" OR "Patella Lead " OR ''Tibial lead'' OR''Calcaneus lead'') \| 4151 \| \| #2 TS=("hypertension" OR ''blood pressure'' OR ''Resistant Hypertension'' OR ''Gestational Hypertension'' OR ''Postpartum Hypertension'' OR ''essential hypertension'' OR ''Secondary Hypertension'' OR ''hypertens*'' OR ''elevat* adj3 blood pressur*'' OR ''high adj3 blood pressur*'') \| \| #3 (TI=(''Cross-sectional'' OR ''Cohort'' OR ''Case-control study '' OR ''Observational Study'' OR ''retrospective study'' OR ''prospective study'')) OR AB=(''Cross-sectional'' OR ''Cohort'' OR ''Case-control study '' OR ''Observational Study'' OR ''retrospective study'' OR ''prospective study'') \| \| #4  TS=((''relative risk'' OR ''RR'' OR ''hazard ratio'' OR ''HR'' OR ''odds ratio'' )) \| \| #5 #1 AND #2 AND #3 AND #4 \|   **Supplementary table3 Retrieval of Embase.**   \| Search via Embase \| Result \| \| --- \| --- \| \| #1 'lead'/exp OR 'lead poisoning'/exp OR 'heavy metals'/exp OR 'heavy metal poisoning'/exp OR 'lead blood level'/exp \| 387 \| \| #2 'blood lead' OR 'urinary lead' OR 'bone lead' OR 'patella lead' OR 'tibial lead' OR 'calcaneus lead':ab,kw,ti \| \| #3 #1 OR #2 \| \| #4 'hypertension'/exp OR 'hypertension' \| \| #5 'blood pressure' OR 'resistant hypertension' OR 'gestational hypertension' OR 'postpartum hypertension' OR 'essential hypertension' OR 'secondary hypertension' OR 'hypertens*' OR 'elevat* adj3 blood pressur*' OR 'high adj3 blood pressur*':ab,kw,ti \| \| #6 #4 OR #5 \| \| #7 'cross-sectional' OR 'cohort' OR 'case-control study' OR 'observational study' OR 'retrospective study' OR 'prospective study':it \| \| #8 'relative risk' OR 'rr' OR 'hazard ratio' OR 'hr' OR 'odds ratio' OR 'or' \| \| #9 #3 AND #6 AND #7 AND #8 \|   .  **Supplementary table4 Retrieval of EBSCO.**   \| Search via EBSCO \| Result \| \| --- \| --- \| \| #1 SU (''lead'' OR ''lead poisoning'' OR '''heavy metals'' OR ''Heavy Metal Poisoning'' OR "Blood lead" OR "Urinary lead" OR "Bone lead" OR "Patella Lead " OR ''Tibial lead'' OR'' Calcaneus lead'') \| 64 \| \| #2 SU ("hypertension" OR ''blood pressure'' OR ''Resistant Hypertension'' OR ''Gestational Hypertension'' OR ''Postpartum Hypertension'' OR ''essential hypertension'' OR ''Secondary Hypertension'' OR ''hypertens*'' OR ''elevat* adj3 blood pressur*'' OR ''high adj3 blood pressur*'') \| \| #3 TX (''relative risk'' OR ''RR'' OR ''hazard ratio'' OR ''HR'' OR ''odds ratio'' OR ''OR'') \| \| #4 #1 AND #2 AND #3 \| \| #5 TI (''Cross-sectional'' OR ''Cohort'' OR ''Case-control study '' OR ''Observational Study'' OR ''retrospective study'' OR ''prospective study'') \| \| #6 AB (''Cross-sectional'' OR ''Cohort'' OR ''Case-control study '' OR ''Observational Study'' OR ''retrospective study'' OR ''prospective study'') \| \| #7 #5 OR #6 \| \| #8 #4 AND #7 \|   **Supplementary table5 Retrieval of Scopus.**   \| Search via Scopus \| Result \| \| --- \| --- \| \| TITLE-ABS-KEY(lead OR lead poisoning OR Blood lead OR Urinary lead OR Bone lead OR Patella Lead OR Tibial lead OR Calcaneus lead) AND TITLE-ABS-KEY(hypertension OR blood pressure OR Resistant Hypertension OR Gestational Hypertension OR Postpartum Hypertension OR essential hypertension OR Secondary Hypertension) AND TITLE-ABS(Cross-sectional OR Cohort OR Case-control study OR Observational Study OR retrospective study OR prospective study) AND ALL(relative risk OR RR OR hazard ratio OR HR OR odds ratio) \| 44 \|   **Supplementary table6 The OR value of the highest quantile of lead exposure.**   \| **S**tudy \| **OR** \| **LCI** \| **UCI** \| \| --- \| --- \| --- \| --- \| \| Michael Rabinowttz 1987 \| 2.5 \| 1.5 \| 3.5 \| \| Iman Al-Saleh 2006 (Blood lead quartiles) \| 0.92 \| 0.41 \| 2.09 \| \| Iman Al-Saleh 2006 (Blood lead levels) \| 1.27 \| 0.71 \| 2.26 \| \| Chadi Yazbeck 2009 \| 2.56 \| 1.05 \| 6.22 \| \| Byung-Kook Lee 2016 (Women ) \| 1.26 \| 0.999 \| 1.58 \| \| Byung-Kook Lee 2016 (Men ) \| 0.88 \| 0.72 \| 1.07 \| \| Angela Gambelunghe 2016 (Men ) \| 1.2 \| 0.96 \| 1.5 \| \| Angela Gambelunghe 2016 (Women ) \| 1.4 \| 1.1 \| 1.7 \| \| Angela Gambelunghe 2016 (Ever-smokers ) \| 1.5 \| 1.2 \| 1.8 \| \| Angela Gambelunghe 2016 (Never-smokers ) \| 0.96 \| 0.7 \| 1.3 \| \| Angela Gambelunghe 2016 (≤57 years ) \| 1.5 \| 1.2 \| 1.9 \| \| Angela Gambelunghe 2016 (＞57 year ) \| 1.3 \| 0.9 \| 1.4 \| \| Alexander R 2018 (Tibia leab ) \| 1.19 \| 1.01 \| 1.41 \| \| Alexander R 2018 (Patella leab ) \| 1.1 \| 0.92 \| 1.31 \| \| Alexander R 2018 (Blood leab ) \| 1.11 \| 0.88 \| 1.4 \| \| Tiange Liu 2019 \| 0.9 \| 0.48 \| 1.68 \| \| Min Gi Kim 2019 \| 1.54 \| 1.26 \| 1.89 \| \| [Hui Miao 2020 (Men )](" \l "jah35177-cr-0001) \| 1.48 \| 1.28 \| 1.71 \| \| [Hui Miao 2020 (Women )](" \l "jah35177-cr-0001) \| 1.316 \| 1.08 \| 1.603 \| \| Su Zhen Wu 2021 (Severe Preeclampsia ) \| 1.12 \| 0.38 \| 3.27 \| \| Su Zhen Wu 2021 (Mild Preeclampsia ) \| 4.26 \| 1.41 \| 12.86 \| \| Hai Duc Nguyen 2021 (Premenopausal women ) \| 5.23 \| 0.45 \| 61.41 \| \| Hai Duc Nguyen 2021 (Postmenopausal women ) \| 1.37 \| 0.43 \| 4.38 \| \| Ziyao Huang 2022 \| 1.12 \| 1.03 \| 1.22 \| \| [Songfeng Zhao 2023](" \l "auth-Songfeng-Zhao-Aff1) \| 0.61 \| 0.45 \| 0.82 \| \| [Yuqing Huang 2023](" \l "auth-Songfeng-Zhao-Aff1) \| 1.26 \| 1.1 \| 1.45 \| \| [Jeoung A Kwon 2023 (Inactive )](" \l "auth-Jeoung_A-Kwon-Aff1-Aff2) \| 1.75 \| 1.29 \| 2.37 \| \| [Jeoung A Kwon 2023 (Moderate active )](" \l "auth-Jeoung_A-Kwon-Aff1-Aff2) \| 2.2 \| 1.36 \| 3.58 \| \| [Jeoung A Kwon 2023 (Most active )](" \l "auth-Jeoung_A-Kwon-Aff1-Aff2) \| 2.08 \| 0.88 \| 4.92 \| \| [Hao Chen 2023 (Men RHTN vs. NRHTN )](https://www.jstage.jst.go.jp/search/global/_search/-char/en?item=8&word=Hao+Chen) \| 1.33 \| 1.01 \| 2.29 \| \| [Hao Chen 2023 (Men RHTN vs. NHTN )](https://www.jstage.jst.go.jp/search/global/_search/-char/en?item=8&word=Hao+Chen) \| 2.45 \| 1.29 \| 4.65 \| \| [Hao Chen 2023 (Men NRHTN vs. NHTN )](https://www.jstage.jst.go.jp/search/global/_search/-char/en?item=8&word=Hao+Chen) \| 1.93 \| 1.58 \| 2.35 \| \| [Hao Chen 2023 (Women RHTN vs. NRHTN )](https://www.jstage.jst.go.jp/search/global/_search/-char/en?item=8&word=Hao+Chen) \| 1.23 \| 0.79 \| 1.91 \| \| [Hao Chen 2023 (Women RHTN vs. NHTN )](https://www.jstage.jst.go.jp/search/global/_search/-char/en?item=8&word=Hao+Chen) \| 2.78 \| 1.71 \| 4.52 \| \| [Hao Chen 2023 (Women NRHTN vs. NHTN )](https://www.jstage.jst.go.jp/search/global/_search/-char/en?item=8&word=Hao+Chen) \| 2.26 \| 1.85 \| 2.77 \| \| [Hao Chen 2023 (≤60years RHTN vs. NRHTN )](https://www.jstage.jst.go.jp/search/global/_search/-char/en?item=8&word=Hao+Chen) \| 1.12 \| 1 \| 1.37 \| \| [Hao Chen 2023 (≤60years RHTN vs. NHTN )](https://www.jstage.jst.go.jp/search/global/_search/-char/en?item=8&word=Hao+Chen) \| 1.55 \| 1.26 \| 1.9 \| \| [Hao Chen 2023 (≤60years NRHTN vs. NHTN )](https://www.jstage.jst.go.jp/search/global/_search/-char/en?item=8&word=Hao+Chen) \| 1.38 \| 1.3 \| 1.47 \| \| [Hao Chen 2023 (＞60years RHTN vs. NRHTN )](https://www.jstage.jst.go.jp/search/global/_search/-char/en?item=8&word=Hao+Chen) \| 1.33 \| 1.1 \| 1.97 \| \| [Hao Chen 2023 (＞60years RHTN vs. NHTN )](https://www.jstage.jst.go.jp/search/global/_search/-char/en?item=8&word=Hao+Chen) \| 1.15 \| 0.75 \| 1.79 \| \| [Hao Chen 2023 (＞60years NRHTN vs. NHTN )](https://www.jstage.jst.go.jp/search/global/_search/-char/en?item=8&word=Hao+Chen) \| 0.87 \| 0.67 \| 1.12 \| \| [Hao Chen 2023 (Kidneydysfunction RHTN vs. NRHTN )](https://www.jstage.jst.go.jp/search/global/_search/-char/en?item=8&word=Hao+Chen) \| 1.7 \| 1.03 \| 2.83 \| \| [Hao Chen 2023 (Kidneydysfunction RHTN vs. NHTN )](https://www.jstage.jst.go.jp/search/global/_search/-char/en?item=8&word=Hao+Chen) \| 2.93 \| 1.66 \| 5.16 \| \| [Hao Chen 2023 (Kidneydysfunction NRHTN vs. NHTN )](https://www.jstage.jst.go.jp/search/global/_search/-char/en?item=8&word=Hao+Chen) \| 1.72 \| 1.29 \| 2.29 \| \| [Hao Chen 2023 (Nokidneydysfunction RHTN vs. NRHTN )](https://www.jstage.jst.go.jp/search/global/_search/-char/en?item=8&word=Hao+Chen) \| 0.98 \| 0.62 \| 1.57 \| \| [Hao Chen 2023 (Nokidneydysfunction RHTN vs. NHTN )](https://www.jstage.jst.go.jp/search/global/_search/-char/en?item=8&word=Hao+Chen) \| 2.19 \| 1.36 \| 3.53 \| \| [Hao Chen 2023 (Nokidneydysfunction NRHTN vs. NHTN )](https://www.jstage.jst.go.jp/search/global/_search/-char/en?item=8&word=Hao+Chen) \| 2.23 \| 1.88 \| 2.63 \| \| Arturo Corbaton Anchuelo 2024 \| 3.4 \| 0.8 \| 14.1 \| \| Cuixiao Wang 2024 \| 1.224 \| 1.176 \| 1.272 \| |  |  |  |
| --- | --- | --- | --- | --- | --- | --- | --- | --- | --- | --- | --- | --- | --- | --- | --- | --- | --- | --- | --- | --- | --- | --- | --- | --- | --- | --- | --- | --- | --- | --- | --- | --- | --- | --- | --- | --- | --- | --- | --- | --- | --- | --- | --- | --- | --- | --- | --- | --- | --- | --- | --- | --- | --- | --- | --- | --- | --- | --- | --- | --- | --- | --- | --- | --- | --- | --- | --- | --- | --- | --- | --- | --- | --- | --- | --- | --- | --- | --- | --- | --- | --- | --- | --- | --- | --- | --- | --- | --- | --- | --- | --- | --- | --- | --- | --- | --- | --- | --- | --- | --- | --- | --- | --- | --- | --- | --- | --- | --- | --- | --- | --- | --- | --- | --- | --- | --- | --- | --- | --- | --- | --- | --- | --- | --- | --- | --- | --- | --- | --- | --- | --- | --- | --- | --- | --- | --- | --- | --- | --- | --- | --- | --- | --- | --- | --- | --- | --- | --- | --- | --- | --- | --- | --- | --- | --- | --- | --- | --- | --- | --- | --- | --- | --- | --- | --- | --- | --- | --- | --- | --- | --- | --- | --- | --- | --- | --- | --- | --- | --- | --- | --- | --- | --- | --- | --- | --- | --- | --- | --- | --- | --- | --- | --- | --- | --- | --- | --- | --- | --- | --- | --- | --- | --- | --- | --- | --- | --- | --- | --- | --- | --- | --- | --- | --- | --- | --- | --- | --- | --- | --- | --- | --- | --- | --- | --- | --- | --- | --- | --- | --- | --- | --- | --- | --- | --- | --- | --- | --- |

**Supplementary table7 Dose-response relationship between different blood lead levels and the risk of hypertension**.

| **Blood lead dose** | **OR** | **LCI** | **UCI** |
| --- | --- | --- | --- |
| 1.107 | 1.16 | 1.31 | 1.04 |
| 2.107 | 1.35 | 1.67 | 1.21 |
| 3.107 | 1.65 | 2.18 | 1.26 |
| 4.107 | 2.03 | 2.88 | 1.42 |
| 5.107 | 2.52 | 4.03 | 1.57 |
| 6.107 | 3.03 | 5.35 | 1.72 |
| 7.107 | 3.81 | 7.82 | 1.88 |
| 8.107 | 4.59 | 10.37 | 2.06 |

**Supplementary table8 NOS quality score included in the case control study.**

| **Study** | **Selection** | | | | **Comparability** | **Outcome** | | | |
| --- | --- | --- | --- | --- | --- | --- | --- | --- | --- |
|  | **Definition of Cases** | **Representativeness of Cases** | **Selection of Controls** | **Definition of Controls** |  | **Ascertainment of Exposure** | **Same Methods of Ascertainment for cases and controls** | **Non-response rate** | **Total** |
| Howard Hu 1996 | 1 | 1 | 1 | 1 | 2 | 1 | 1 | 1 | 9 |
| Iman Al-Saleh 2006 | 1 | 1 | 1 | 1 | 0 | 1 | 1 | 1 | 7 |

**Supplementary table9 NOS quality score included in the cohort study.**

| **Study** | **Selection** | | | | **Comparability** | **Outcome** | | | **Summary** |
| --- | --- | --- | --- | --- | --- | --- | --- | --- | --- |
|  | **Representativeness of the exposed cohort** | **Selection of the non-exposed cohort** | **Ascertainment of exposure** | **Demonstration that outcome of interest was not present at start of study** |  | **Assessment of outcome** | **Was follow-up long enough for outcomes to occur** | **Adequacy of follow up of cohorts** |  |
| Stephen J. Rothenberg 2002 | 1 | 0 | 1 | 1 | 2 | 1 | 1 | 0 | 7 |
| *Chadi Yazbeck 2009* | 1 | 0 | 1 | 1 | 2 | 1 | 1 | 1 | 8 |
| Angela Gambelunghe 2016 | 1 | 0 | 1 | 1 | 2 | 1 | 1 | 1 | 8 |
| Alexander R 2018 | 1 | 0 | 1 | 0 | 2 | 1 | 1 | 1 | 7 |
| Tiange Liu 2019 | 1 | 0 | 1 | 1 | 2 | 1 | 1 | 1 | 8 |
| Min Gi Kim 2019 | 1 | 0 | 1 | 0 | 0 | 1 | 1 | 0 | 5 |
| Su Zhen Wu 2021 | 1 | 0 | 1 | 1 | 2 | 1 | 1 | 1 | 8 |

**Supplementary table10 JBI scale score included in cross-sectional studies.**

| **Study** | **v1** | **v2** | **v3** | **v4** | **v5** | **v6** | **v7** | **v8** | **v9** | **Summary** |
| --- | --- | --- | --- | --- | --- | --- | --- | --- | --- | --- |
| W.L.A.M. de Kort 1987 | 1 | 1 | 1 | 1 | 1 | 1 | 1 | 1 | 1 | 9 |
| MICHAEL RABINOWTTZ 1987 | 1 | 0 | 1 | 1 | 1 | 0 | 0 | 1 | 0 | 5 |
| David Martin 2006 | 1 | 1 | 1 | 1 | 1 | 1 | 1 | 1 | 0 | 8 |
| Byung-Kook Lee 2016 | 1 | 1 | 1 | 1 | 1 | 1 | 1 | 1 | 0 | 8 |
| Wen-Yi Yang 2018 | 1 | 0 | 1 | 1 | 1 | 1 | 1 | 1 | 0 | 7 |
| Katherine M. Johnson 2020 | 1 | 0 | 1 | 1 | 1 | 1 | 1 | 1 | 0 | 7 |
| [Hui Miao 2020](" \l "jah35177-cr-0001) | 1 | 1 | 1 | 1 | 1 | 1 | 1 | 1 | 0 | 8 |
| Hai Duc Nguyen 2021 | 1 | 1 | 1 | 1 | 1 | 1 | 1 | 1 | 0 | 8 |
| Ziyao Huang 2022 | 1 | 1 | 1 | 1 | 0 | 1 | 1 | 1 | 0 | 7 |
| [Jeoung A Kwon 2023](" \l "auth-Jeoung_A-Kwon-Aff1-Aff2) | 1 | 1 | 1 | 1 | 1 | 1 | 1 | 1 | 0 | 8 |
| [Songfeng Zhao 2023](" \l "auth-Songfeng-Zhao-Aff1) | 1 | 1 | 1 | 1 | 1 | 1 | 1 | 1 | 0 | 8 |
| [Yuqing Huang 2023](" \l "auth-Songfeng-Zhao-Aff1) | 1 | 1 | 1 | 1 | 0 | 1 | 1 | 1 | 0 | 7 |
| [Hao Chen 2023](https://www.jstage.jst.go.jp/search/global/_search/-char/en?item=8&word=Hao+Chen) | 1 | 1 | 1 | 1 | 0 | 1 | 1 | 1 | 0 | 7 |
| Arturo Corbaton ´ Anchuelo 2024 | 1 | 1 | 0 | 1 | 0 | 1 | 1 | 1 | 0 | 6 |
| Cuixiao Wang 2024 | 1 | 1 | 1 | 1 | 0 | 1 | 1 | 1 | 0 | 7 |

Notes: 1 = The maximum score is 9, with higher scores indicating higher research quality. Each item can score up to 1 point.

2 = When using the JBI scale for quality assessment of cross-sectional studies, V1 = Was the sample frame appropriate to address the target population? V2 = Were study participants sampled in an appropriate way? V3 = Was the sample size adequate? V4 = Were the study subjects and the setting described in detail? V5 = Was the data analysis conducted with sufficient coverage of the identified sample. V6 = Were valid methods used for the identification of the condition? V7 = Was the condition measured in a standard, reliable way for all participants? V8 = Was there appropriate statistical analysis? V9 = Was the response rate adequate, and if not, was the low response rate managed appropriately?

Abbreviation: JBI, Journal of Biomedical Informatics

**Supplementary Table 11 PRISMA 2020 statement.**

| **Section and Topic** | **Item #** | **Checklist item** | **Location where item is reported** |
| --- | --- | --- | --- |
| **TITLE** | | |  |
| Title | 1 | Identify the report as a systematic review. | 1 |
| **ABSTRACT** | | |  |
| Abstract | 2 | See the PRISMA 2020 for Abstracts checklist. | 1 |
| **INTRODUCTION** | | |  |
| Rationale | 3 | Describe the rationale for the review in the context of existing knowledge. | 2-3 |
| Objectives | 4 | Provide an explicit statement of the objective(s) or question(s) the review addresses. | 4 |
| **METHODS** | | |  |
| Eligibility criteria | 5 | Specify the inclusion and exclusion criteria for the review and how studies were grouped for the syntheses. | 4 |
| Information sources | 6 | Specify all databases, registers, websites, organisations, reference lists and other sources searched or consulted to identify studies. Specify the date when each source was last searched or consulted. | 4 |
| Search strategy | 7 | Present the full search strategies for all databases, registers and websites, including any filters and limits used. | 4 |
| Selection process | 8 | Specify the methods used to decide whether a study met the inclusion criteria of the review, including how many reviewers screened each record and each report retrieved, whether they worked independently, and if applicable, details of automation tools used in the process. | 5 |
| Data collection process | 9 | Specify the methods used to collect data from reports, including how many reviewers collected data from each report, whether they worked independently, any processes for obtaining or confirming data from study investigators, and if applicable, details of automation tools used in the process. | 5 |
| Data items | 10a | List and define all outcomes for which data were sought. Specify whether all results that were compatible with each outcome domain in each study were sought (e.g. for all measures, time points, analyses), and if not, the methods used to decide which results to collect. | 5-6 |
|  | 10b | List and define all other variables for which data were sought (e.g. participant and intervention characteristics, funding sources). Describe any assumptions made about any missing or unclear information. | 6 |
| Study risk of bias assessment | 11 | Specify the methods used to assess risk of bias in the included studies, including details of the tool(s) used, how many reviewers assessed each study and whether they worked independently, and if applicable, details of automation tools used in the process. | 6 |
| Effect measures | 12 | Specify for each outcome the effect measure(s) (e.g. risk ratio, mean difference) used in the synthesis or presentation of results. | 6 |
| Synthesis methods | 13a | Describe the processes used to decide which studies were eligible for each synthesis (e.g. tabulating the study intervention characteristics and comparing against the planned groups for each synthesis (item #5)). | 6 |
|  | 13b | Describe any methods required to prepare the data for presentation or synthesis, such as handling of missing summary statistics, or data conversions. | 6 |
|  | 13c | Describe any methods used to tabulate or visually display results of individual studies and syntheses. | 6 |
|  | 13d | Describe any methods used to synthesize results and provide a rationale for the choice(s). If meta-analysis was performed, describe the model(s), method(s) to identify the presence and extent of statistical heterogeneity, and software package(s) used. | 6 |
|  | 13e | Describe any methods used to explore possible causes of heterogeneity among study results (e.g. subgroup analysis, meta-regression). | 6-7 |
|  | 13f | Describe any sensitivity analyses conducted to assess robustness of the synthesized results. | 6-7 |
| Reporting bias assessment | 14 | Describe any methods used to assess risk of bias due to missing results in a synthesis (arising from reporting biases). | 7 |
| Certainty assessment | 15 | Describe any methods used to assess certainty (or confidence) in the body of evidence for an outcome. | 7 |
| **RESULTS** | | |  |
| Study selection | 16a | Describe the results of the search and selection process, from the number of records identified in the search to the number of studies included in the review, ideally using a flow diagram. | 7 |
|  | 16b | Cite studies that might appear to meet the inclusion criteria, but which were excluded, and explain why they were excluded. | 7 |
| Study characteristics | 17 | Cite each included study and present its characteristics. | 7 |
| Risk of bias in studies | 18 | Present assessments of risk of bias for each included study. | 7 |
| Results of individual studies | 19 | For all outcomes, present, for each study: (a) summary statistics for each group (where appropriate) and (b) an effect estimate and its precision (e.g. confidence/credible interval), ideally using structured tables or plots. | 7 |
| Results of syntheses | 20a | For each synthesis, briefly summarise the characteristics and risk of bias among contributing studies. | 8 |
|  | 20b | Present results of all statistical syntheses conducted. If meta-analysis was done, present for each the summary estimate and its precision (e.g. confidence/credible interval) and measures of statistical heterogeneity. If comparing groups, describe the direction of the effect. | 8 |
|  | 20c | Present results of all investigations of possible causes of heterogeneity among study results. | 8 |
|  | 20d | Present results of all sensitivity analyses conducted to assess the robustness of the synthesized results. | 8 |
| Reporting biases | 21 | Present assessments of risk of bias due to missing results (arising from reporting biases) for each synthesis assessed. | 9 |
| Certainty of evidence | 22 | Present assessments of certainty (or confidence) in the body of evidence for each outcome assessed. | 9 |
| **DISCUSSION** | | |  |
| Discussion | 23a | Provide a general interpretation of the results in the context of other evidence. | 10 |
|  | 23b | Discuss any limitations of the evidence included in the review. | 11 |
|  | 23c | Discuss any limitations of the review processes used. | 11 |
|  | 23d | Discuss implications of the results for practice, policy, and future research. | 11 |
| **OTHER INFORMATION** | | |  |
| Registration and protocol | 24a | Provide registration information for the review, including register name and registration number, or state that the review was not registered. | 4 |
|  | 24b | Indicate where the review protocol can be accessed, or state that a protocol was not prepared. | 4 |
|  | 24c | Describe and explain any amendments to information provided at registration or in the protocol. | 4 |
| Support | 25 | Describe sources of financial or non-financial support for the review, and the role of the funders or sponsors in the review. | 11 |
| Competing interests | 26 | Declare any competing interests of review authors. | 12 |
| Availability of data, code and other materials | 27 | Report which of the following are publicly available and where they can be found: template data collection forms; data extracted from included studies; data used for all analyses; analytic code; any other materials used in the review. | 12 |

*From:*  Page MJ, McKenzie JE, Bossuyt PM, Boutron I, Hoffmann TC, Mulrow CD, et al. The PRISMA 2020 statement: an updated guideline for reporting systematic reviews. BMJ 2021;372:n71. doi: 10.1136/bmj.n71


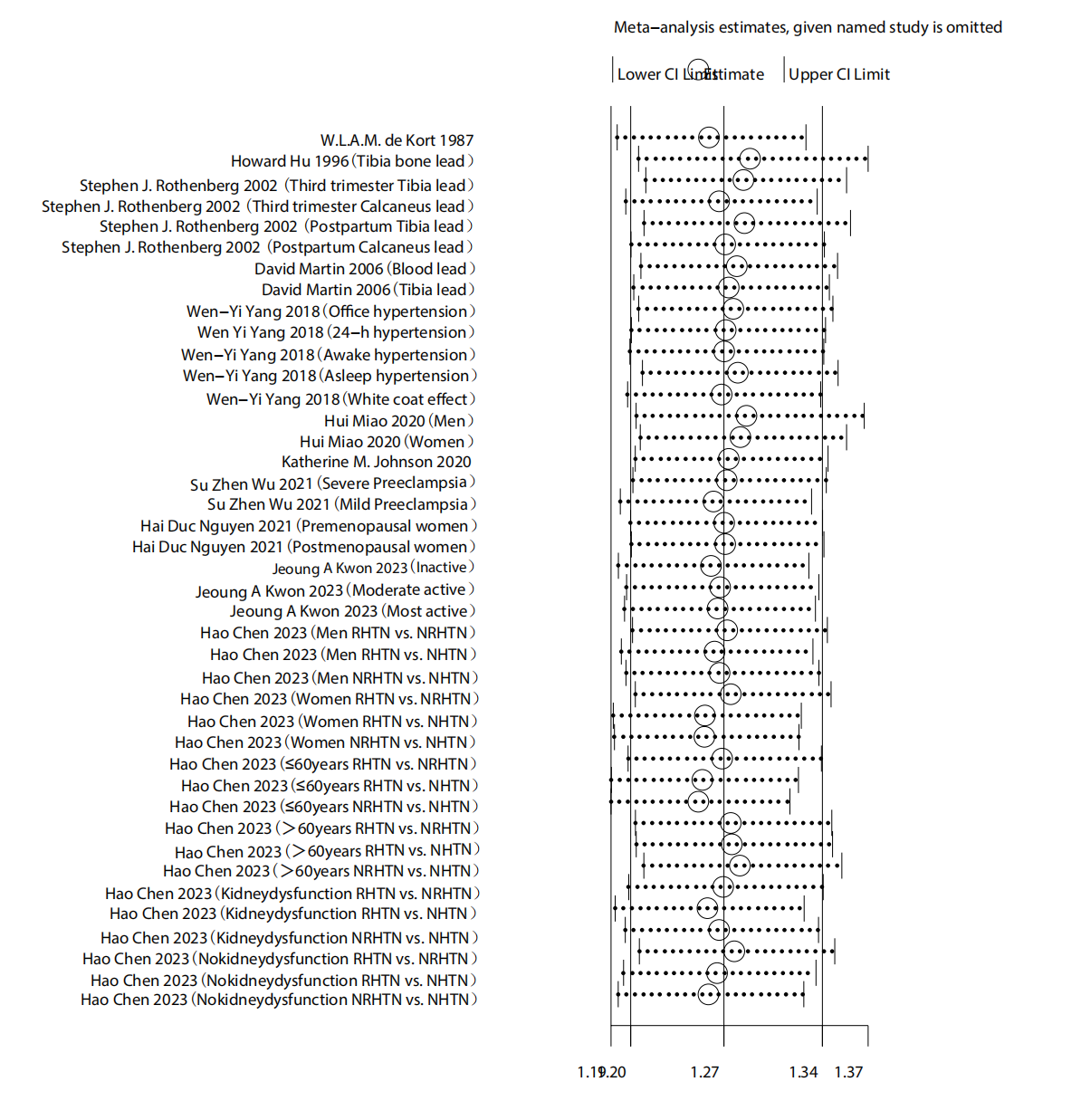


**Supplementary Figure 1 Correlation sensitivity analysis.**


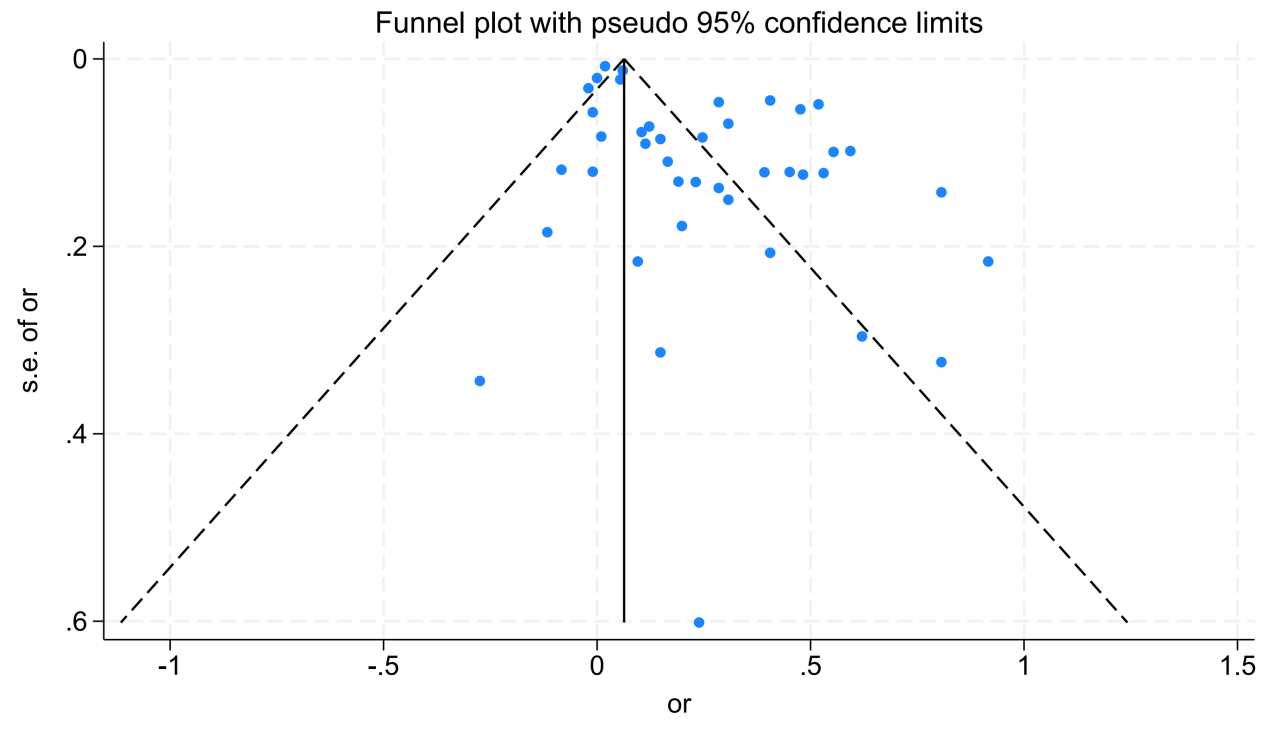


**Supplementary Figure 2 Correlation funnel plot.**


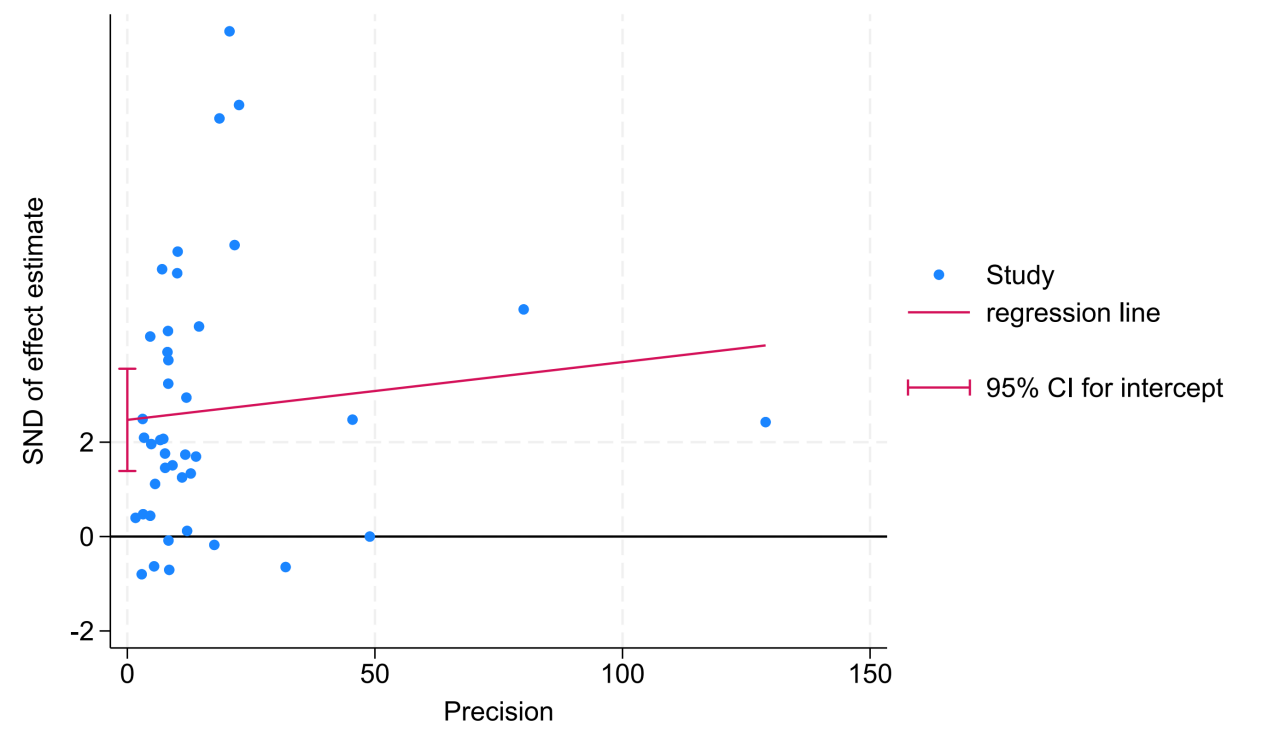


**Supplementary Figure 3 Correlation publication bias.**


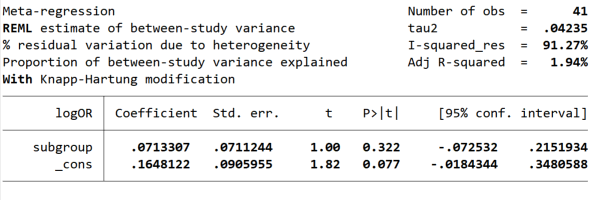


**Supplementary Figure 4 Meta-regression based on country.**


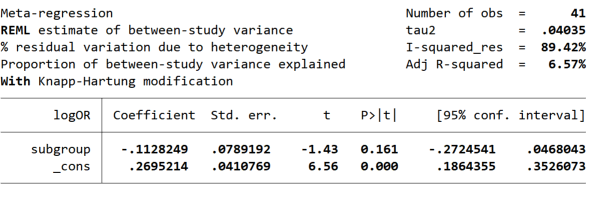


**Supplementary Figure 5 Meta-regression based on study design.**


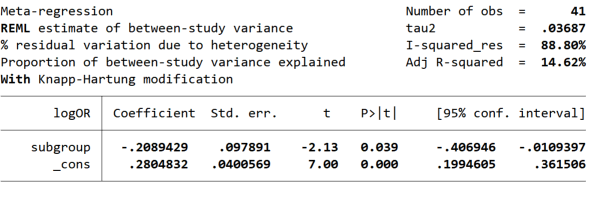


**Supplementary Figure 6 Meta-regression based on lead exposure measurement.**


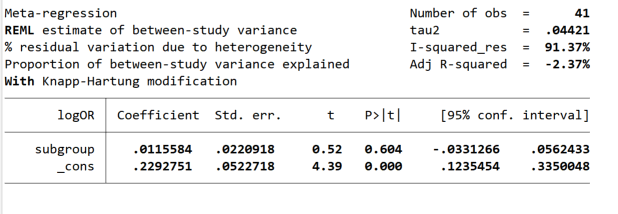
**Supplementary Figure 7 Meta-regression based on hypertension subtype.**


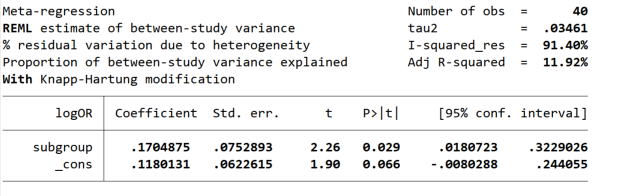


**Supplementary Figure 8 Meta-regression based on drinking adjustment.**


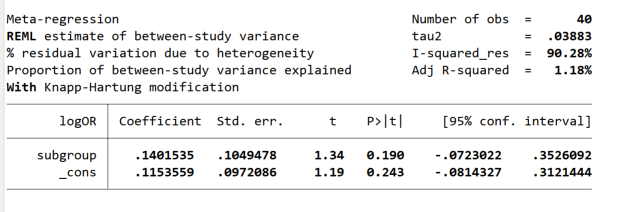


**Supplementary Figure 9 Meta-regression based on smoking adjustment.**


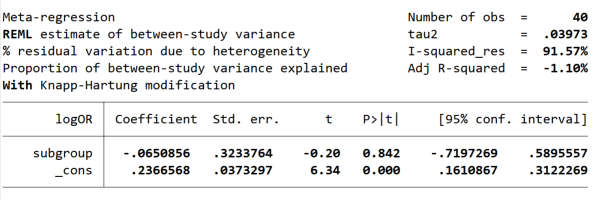


**Supplementary Figure 10 Meta-regression based on physical-activity adjustment.**


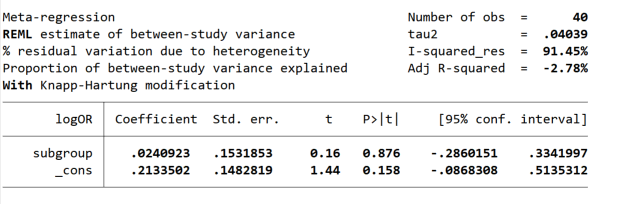


**Supplementary Figure 11 Meta-regression based on BMI adjustment.**


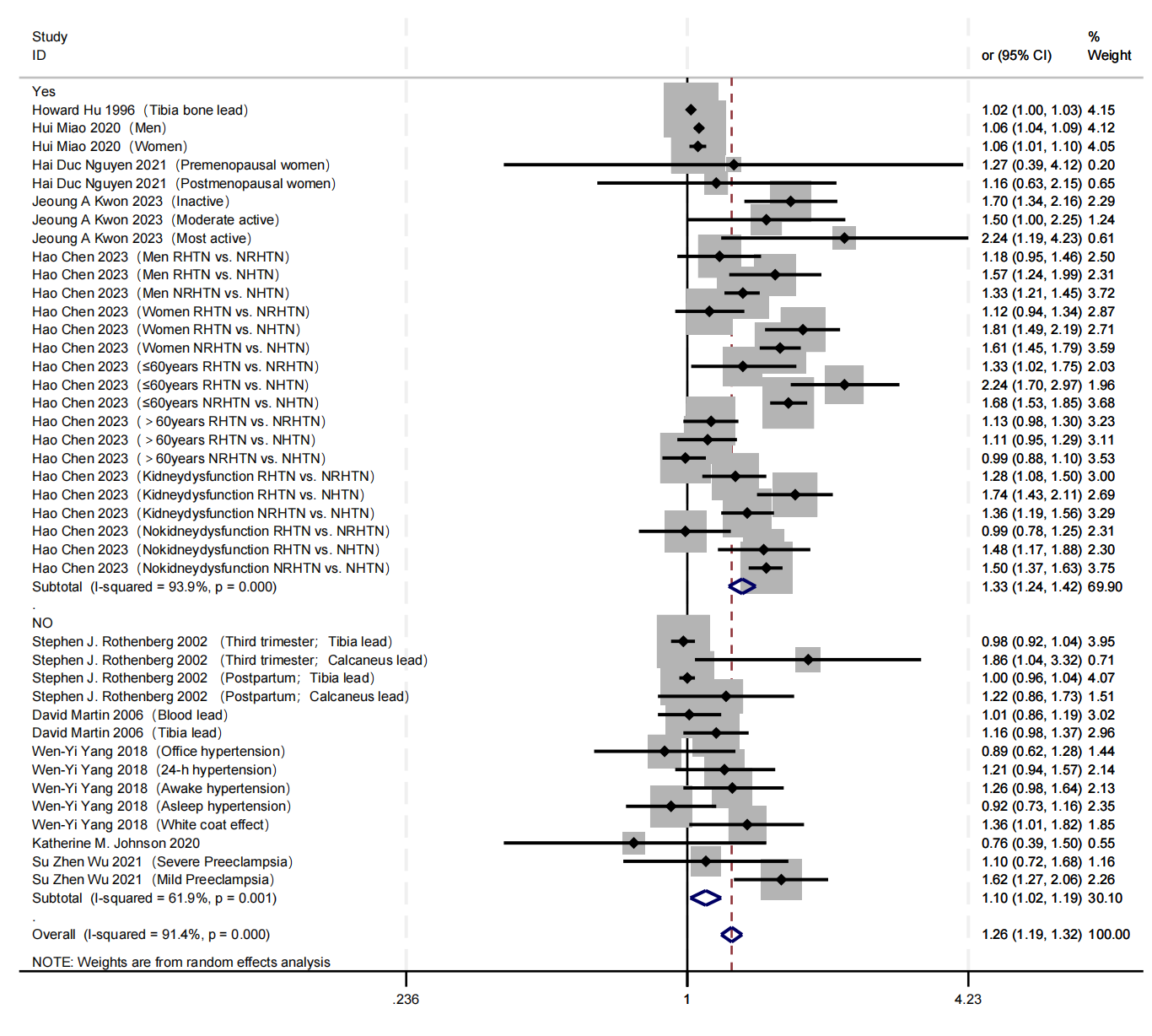


**Supplementary Figure 12 Subgroup analysis based on drinking adjustment (adjusted vs. unadjusted).**


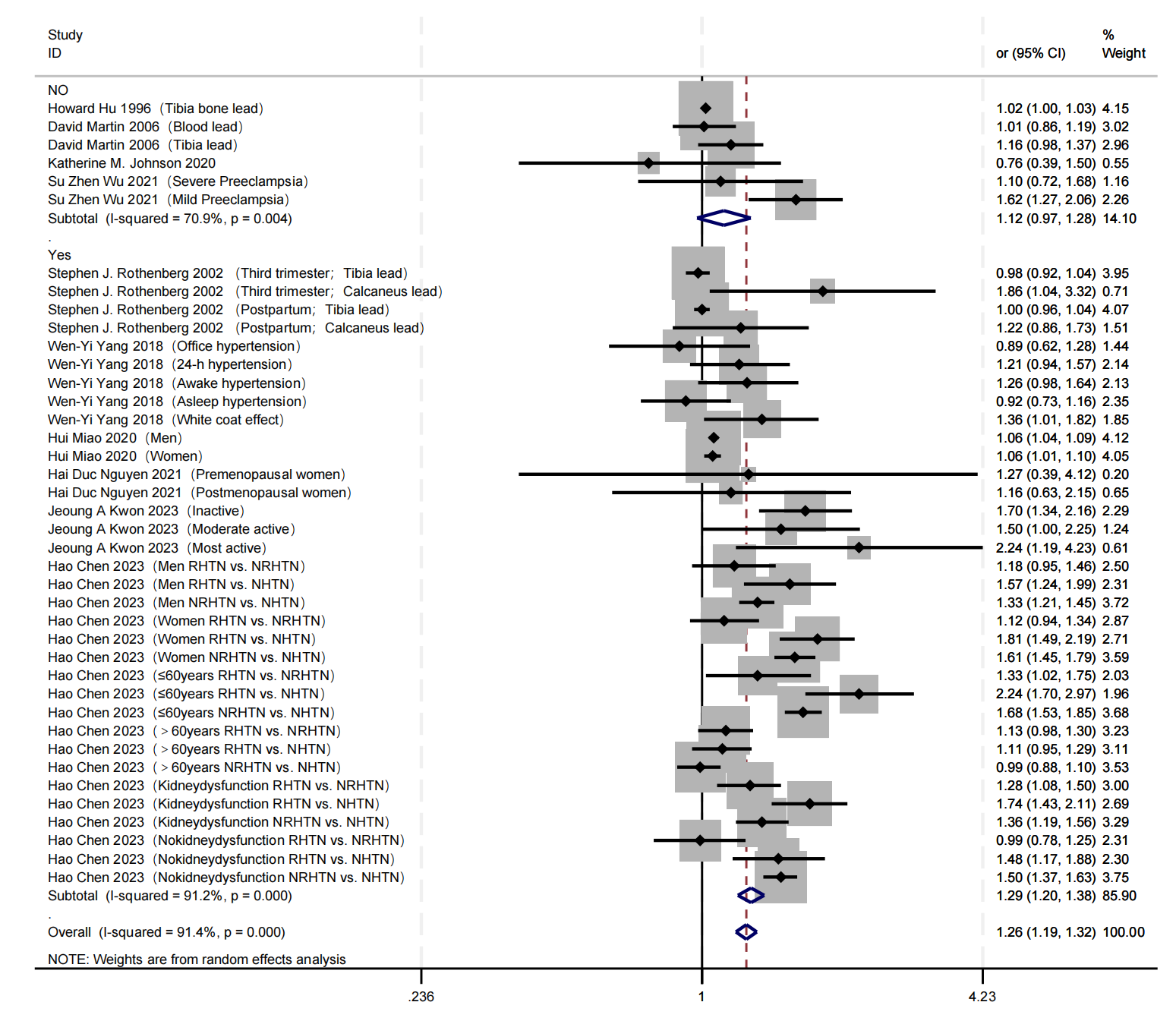


**Supplementary Figure 13 Subgroup analysis based on smoking adjustment (adjusted vs. unadjusted).**


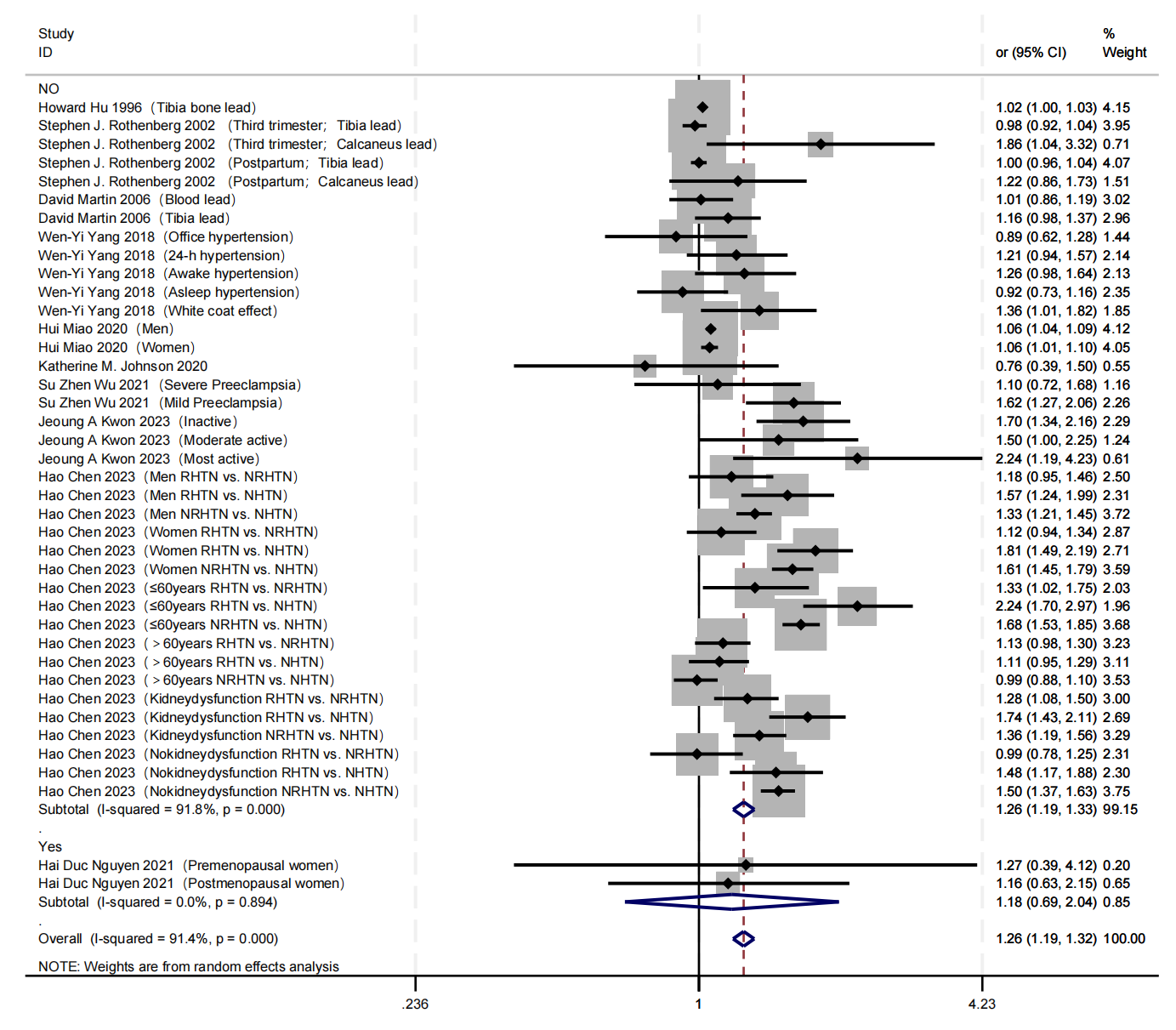


**Supplementary Figure 14 Subgroup analysis based on physical-activity adjustment (adjusted vs. unadjusted).**


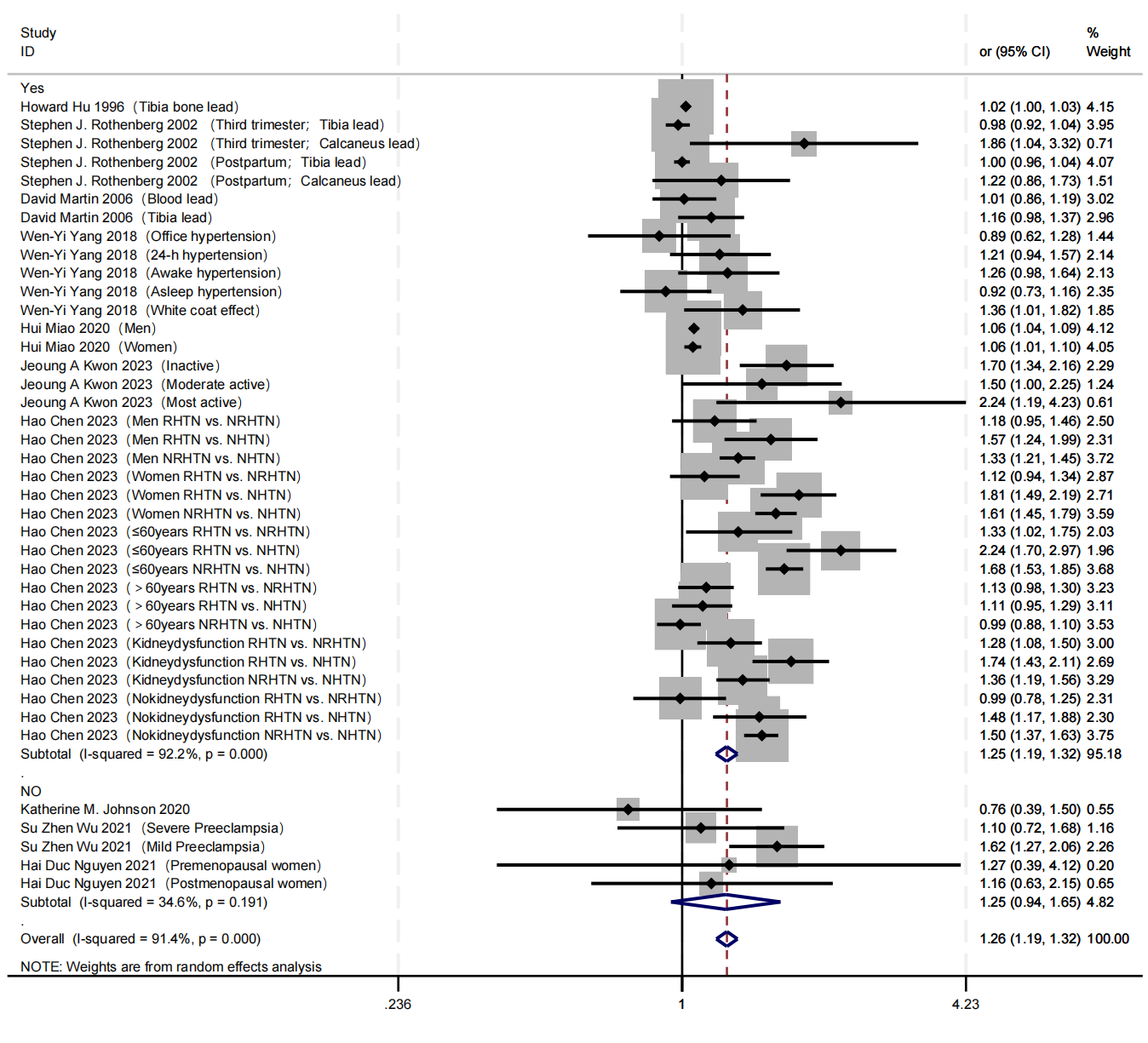


**Supplementary Figure 15 Subgroup analysis based on BMI adjustment (adjusted vs. unadjusted).**


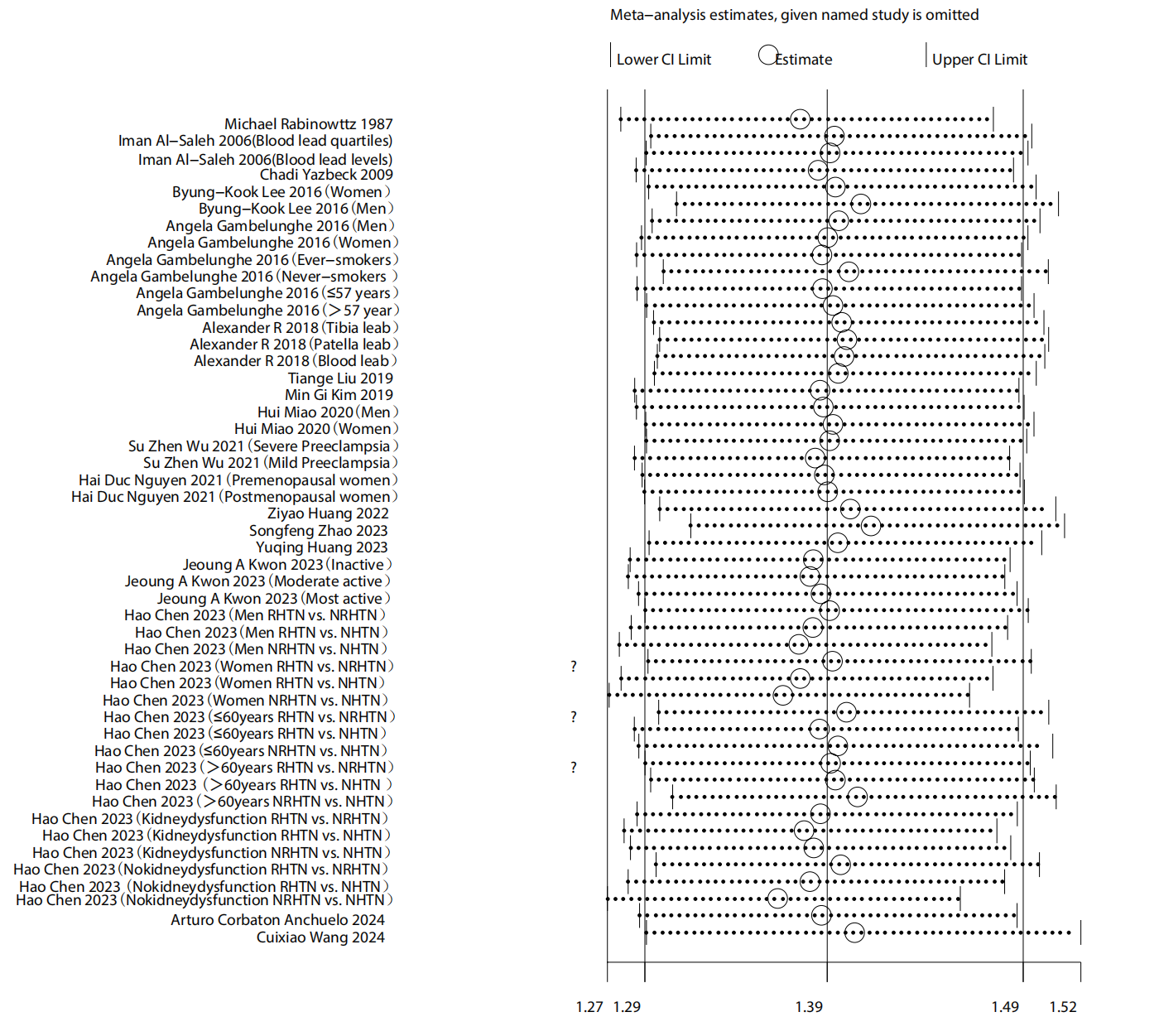


**Supplementary Figure 16 The highest measurement sensitivity analysis.**


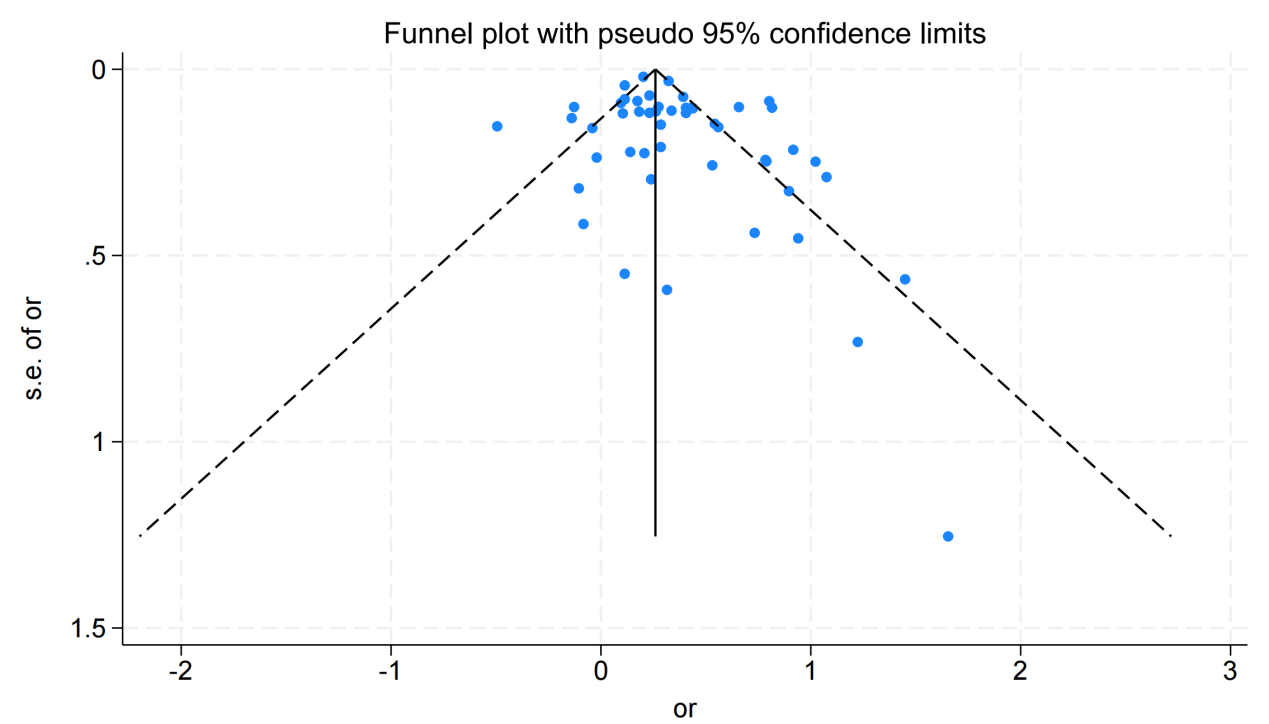


**Supplementary Figure 17 The highest dose funnel plot.**


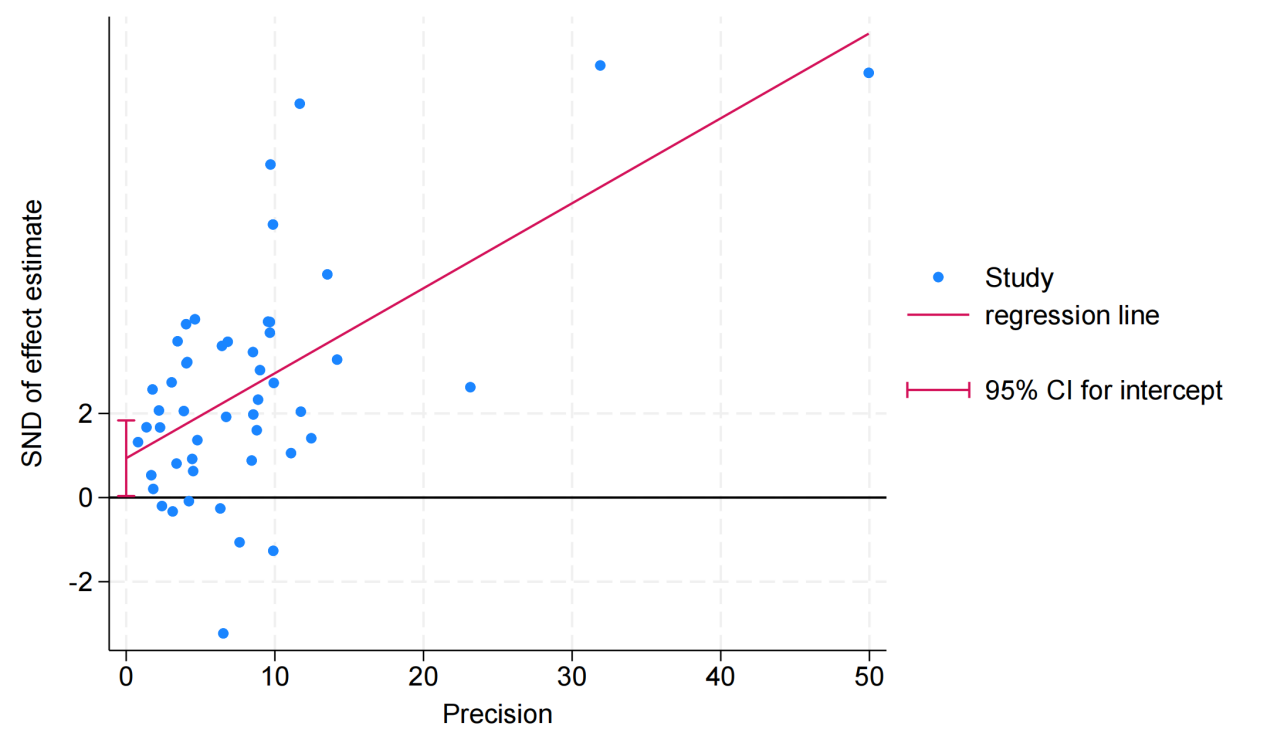
**Supplementary Figure 18 Maximum dose publication bias.**
